# Senolysis induced by 25-hydroxycholesterol targets CRYAB in multiple cell types

**DOI:** 10.1016/j.isci.2022.103848

**Published:** 2022-02-02

**Authors:** Chandani Limbad, Ryosuke Doi, Julia McGirr, Serban Ciotlos, Kevin Perez, Zachary S. Clayton, Radha Daya, Douglas R. Seals, Judith Campisi, Simon Melov

**Affiliations:** 1 Buck Institute for Research on Aging, Novato, CA, USA; 2 Department of Integrative Physiology, University of Colorado Boulder, Boulder, CO, USA; 3 Lawrence Berkeley National Laboratory, Berkeley, CA, USA

**Keywords:** Biological sciences, Molecular biology, Cell biology, Transcriptomics

## Abstract

Cellular senescence is a driver of many age-related pathologies. There is an active search for pharmaceuticals termed senolytics that can mitigate or remove senescent cells *in vivo* by targeting genes that promote the survival of senescent cells. We utilized single-cell RNA sequencing to identify CRYAB as a robust senescence-induced gene and potential target for senolysis. Using chemical inhibitor screening for CRYAB disruption, we identified 25-hydroxycholesterol (25HC), an endogenous metabolite of cholesterol biosynthesis, as a potent senolytic. We then validated 25HC as a senolytic in mouse and human cells in culture and *in vivo* in mouse skeletal muscle. Thus, 25HC represents a potential class of senolytics, which may be useful in combating diseases or physiologies in which cellular senescence is a key driver.

## Highlights

• Single cell sequencing can be used to facilitate discovery of senolytic targets
• CRYAB is a senolytic target in multiple human and mouse cell types
• 25-hydroxycholesterol is a naturally produced senolytic
• 25-hydroxycholesterol senolysis was validated in mice using the biomarker 15-dPGJ2

## Graphical Abstract

**Figure:**
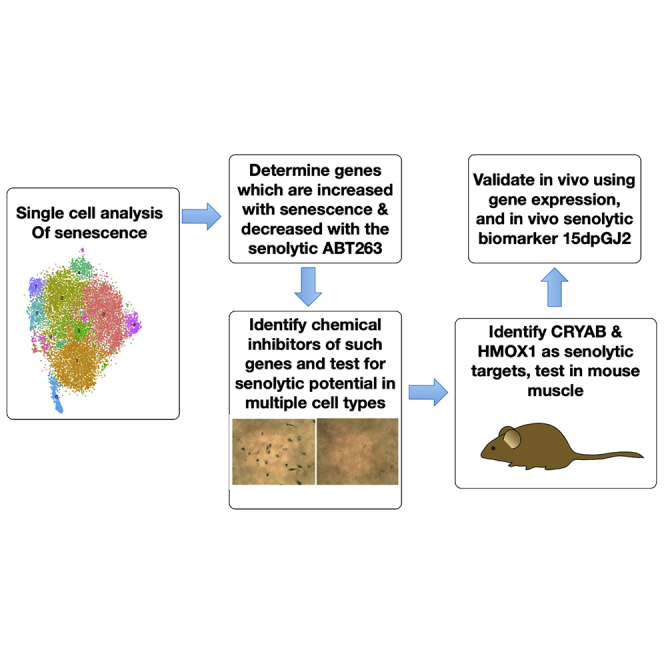

## Introduction

Cellular senescence causes proliferative cells to undergo an essentially permanent cell-cycle arrest upon encountering stressful stimuli such as genotoxic chemotherapy, radiation, or telomere shortening (Bernadotte et al., 2016; Demaria et al., 2017; Limbad et al., 2020). Increasing evidence indicates that the senescent phenotype is not restricted to mitotic cells, but can also affect predominantly postmitotic tissues such as those in brain, skeletal muscle, or heart (Anderson et al., 2019; Sapieha and Mallette, 2018). Senescent cells accumulate with age in many organs and create a pro-inflammatory milieu through a senescence-associated secretory phenotype (SASP) (Rodier and Campisi, 2011). The widely used chemotherapeutic agent Doxorubicin (Doxo) induces senescence in mice and cultured cells (Demaria et al., 2017), and is associated with severe side effects such as cardiotoxicity, muscle weakness, fatigue, and cognitive impairment (Chatterjee et al., 2010; Gilliam and St Clair, 2011; Keeney et al., 2018; van Norren et al., 2009). Genetic ablation of senescent cells in Doxo-treated mice improves function in several tissues (Demaria et al., 2017). Consequently, there is an active search for drugs that can kill senescent cells (senolytics) (van Deursen, 2019). This class of drugs improves health span in multiple contexts of aging, and has potential to mitigate many age related diseases (Chang et al., 2016; Fuhrmann-Stroissnigg et al., 2017; Kim and Kim, 2019; Kirkland and Tchkonia, 2017; Wissler Gerdes et al., 2020; Xu et al., 2018). For example, the senolytic ABT263 (ABT) improves age-associated pathologies, including declines in hematopoiesis and cognition, diabetes, and myocardial infarction (Aguayo-Mazzucato et al., 2019; Bussian et al., 2018; Chang et al., 2016; Gonzalez-Gualda et al., 2020; Kirkland and Tchkonia, 2017; Walaszczyk et al., 2019). The potential benefits of ABT as a senolytic were also studied after skeletal muscle injury (Chiche et al., 2017), but no benefits were reported in aging skeletal muscle.

Skeletal muscle is among the largest organs in the human body and provides a means to generate movement and maintain metabolic homeostasis. Muscle regeneration and maintenance are facilitated by resident mesenchymal progenitors and muscle stem cells, also known as fibro-adipogenic progenitors (FAPs), and satellite cells (SCs). Skeletal muscle mass and function decline with aging, culminating in sarcopenia, and is linked to an increased burden of senescent cells (Lukjanenko et al., 2019; Saito et al., 2020; Sousa-Victor et al., 2014). However, senescent cells comprise only a small fraction of cells in tissue. Consequently, it is difficult to identify and target such cells by conventional bulk analyses. Single-cell sequencing and cell identity assignation by transcriptional profiling is now increasingly applied to diverse tissues of heterogeneous cell makeup (Brbic et al., 2020; Fu et al., 2020; Grun et al., 2015; Papalexi and Satija, 2018), including skeletal muscle, heart, and brain (Carter et al., 2018; Rubenstein et al., 2020; Skelly et al., 2018). To identify senolytic targets and putative senolytics focused on such targets, we leveraged the power of single cell profiling using skeletal muscle as a model system to determine transcriptional profiles of senescent cell subpopulations. This approach is particularly useful when defining cells which are present at a low frequency within complex tissues, such as satellite cells or FAPs. Using single cell profiling, we discovered a senolytic and its corresponding target in skeletal muscle, and confirmed the generality and efficacy in multiple diverse cell types of both human and mouse origin.

We performed single-cell RNA sequencing (scRNA seq) on SCs and FAPs from mice treated with vehicle, Doxo, or Doxo + ABT to identify potential senolytic targets. Our results demonstrate that Doxo treatment induces senescence in FAPs and SCs, and that ABT reduces the fraction of both senescent cell types. In addition, using qRT-PCR and shRNA knockdowns, we identified *CRYAB,* a small heat shock protein, and *HMOX1* (heme oxygenase 1) as a common senolytic target in both cell types. Our results show that 25-hydroxycholesterol (25HC), which blocks *CRYAB* aggregation (Makley et al., 2015), is a senolytic drug for FAPs and SCs. Further, we demonstrated the senolytic activity of 25HC in multiple other cell types from human and mouse organs. Finally, we show that 25HC results in senolysis in naturally aged mice or young mice treated with Doxo using the recently discovered *in vivo* biomarker 15days-PGJ2, detectable in urine. Our results suggest that scRNA seq can be used to discover therapeutic targets, inhibitors of *CRYAB* can lead to potent senolysis, and 25HC is a promising senolytic to treat senescence-associated pathologies.

## Results

### scRNA sequencing of senescent and non-senescent FAPs and SCs from mouse skeletal muscle identifies senolytic targets

**Figure 1:**
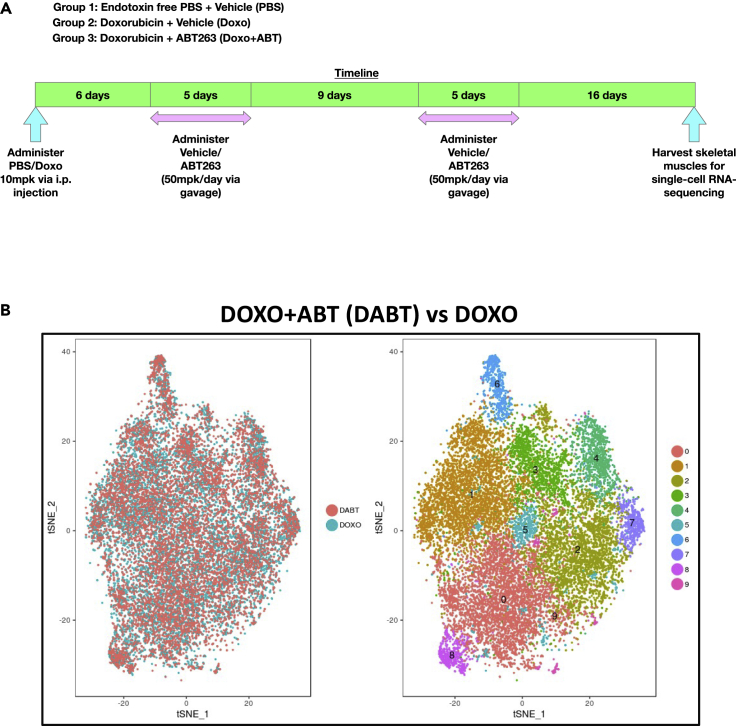
Single-cell RNA sequencing of senescent and non-senescent FAPs and SCs from mouse skeletal muscles identify senolytic target genes (A) *In vivo* experimental groups and timeline. PBS, Doxo, and Doxo + ABT groups were injected with PBS or Doxo. 6 days later, vehicle or ABT was given for five consecutive days and repeated after 9 days 16 days after the second cycle of ABT treatment, hindlimb skeletal muscles were isolated from all three groups (n = 5/group). (B) t-SNE plot showing the embeddings (left) and clustering (right) of cells analyzed in FAPs DOXO vs PBS comparisons. 10 distinct clusters are seen in the t-SNE plot

We treated four-month-old male mice with PBS, Doxo (in PBS) or Doxo + ABT (in 10% EtOH, 30% PEG400, 60% Phosal 50 PG; termed vehicle) (Figure 1). Doxo (10 mg/kg) alone induced senescence, whereas mice treated with Doxo and ABT (50 mg/kg/day) had a reduced burden of senescent cells, consistent with prior studies (Chang et al., 2016; Demaria et al., 2017). To identify subpopulations of senescent cells within a complex tissue, we chose skeletal muscle given its importance as a critical largely postmitotic tissue that declines in mass and function with age. As outlined in the schematic in Figure 1A, we harvested bulk skeletal muscle from all treatment groups at the end of the experiment to isolate either FAPs or SCs because of their importance in maintaining tissue function. We used fluorescence-activated cell sorting (FACS) followed by scRNA seq to identify and enumerate cells of interest (Figures 1, S1A, and S1B). FACs analysis showed that FAPs comprised ∼3% of all cells counted, whereas SCs comprised ∼1% of the cell population (Figures 1 and S1B).

We used the sorted FAPs and SCs to create 10X-3′ scRNA-seq libraries for gene expression analysis (Figures 1 and S1C, Table S1). The sequencing data were processed by CellRanger v2.1.0 and Seurat v2.3. CellRanger results demonstrated that we had captured 9-10K FAPs and 6-9K SCs, with a mean read/cell of 12–17K reads, and median genes/cell of 400–1K (Figure 1, Table S2). For both populations, differential expression analyses were performed on Doxo vs PBS libraries and Doxo + ABT vs Doxo libraries. The Seurat workflow was used for filtering, normalization, scaling, canonical correlation analysis, UMAP generation, and clustering (Stuart et al., 2019). Comparing SC libraries from Doxo-treated and PBS-treated samples, 851 and 2,251 genes were significantly upregulated and downregulated, respectively, across all clusters. Comparing SC clusters from Doxo + ABT vs Doxo alone, 1,534 and 914 genes were upregulated and downregulated, respectively. Comparing Doxo and PBS libraries prepared from FAPs, 680 and 678 genes were significantly upregulated and downregulated, respectively. Finally, FAPs treated with Doxo + ABT vs Doxo alone yielded 1,061 and 327 differentially regulated genes, respectively (Figure 1B).

**Figure 2:**
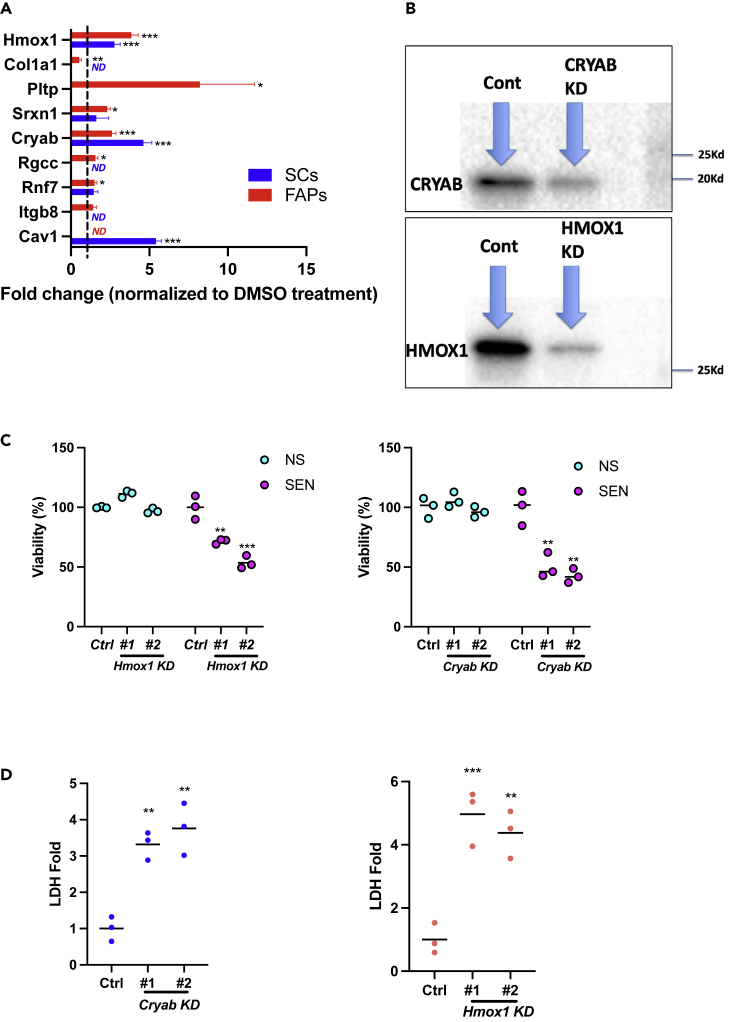
CRYAB and HMOX1 are senolytic targets (A) mRNA levels of selected candidate genes in FAPs (red) and SCs (blue) treated with Doxo. RNA was collected 7 days after replacing media containing Doxo. mRNA was quantified by qRT-PCR, normalized to *actin* mRNA, and values for DMSO-treatment was set to 1 (dashed line). In SCs; Col1a1, Pltp, Rgcc, Itgb8 were not detected (“ND”). In FAPs Cav1 was not detected (“ND”). (B) Confirmation by Western blot of KD of both CRYAB and HMOX1. shRNA of CRYAB or HMOX1 was performed in FAPs using an MOI of 2, and cells collected after 5 days of culture following transfection. Antibodies against either CRYAB (F-10 Santa Cruz) or HMOX1 (E6Z5G, Cell Signaling Technology) were used on protein lysates to determine KD at the level of protein. Equal amounts of protein were loaded as shown by actin normalization. (C) Effects of *Cryab* or *Hmox1* knockdown on the viability of non-senescent (NS) or senescent (SEN) FAPs. Cells were pre-cultured for 24 h, infected with five MOI of lentiviruses expressing control *shRNA* (*Ctrl.*) or two different *shRNAs* (*#1*, *#2)* for each gene, treated with 2 mg/mL puromycin for 3 days and then with 250 nM Doxo or DMSO for 24 h. Cell viability was analyzed at 9 days after replacing media containing Doxo. The average value of *Ctrl* was set at 100%. (D) Extracellular LDH analysis. Supernatants were collected 9 days after removing media containing Doxo. The average value of *Ctrl* was set at 1. p values for DMSO and *Ctrl* were obtained using unpaired two-tailed Student’s t test in (A) or Dunnett's multiple comparison test in (C) and (D). Mean ± SE, n = 3, ∗p < 0.05, ∗∗p < 0.01, ∗∗∗p < 0.001

We reasoned that gene expression positivity for p16^INK4a^ (p16) or p21^CIP1^ (p21), two proteins commonly associated with senescence, might reveal unique subpopulations of senescent cells. We identified specific clusters expressing *Cdkn2A* (encoding p16) or *Cdkn1A* (encoding p21) in libraries from FAPs and SCs (Figure 1, Table S1). To further identify potential senescent subpopulations, we created lists of differentially expressed genes for FAPs and SCs using the following filters: (p value of <0.05, average logFC ≤ −0.25, or ≥0.25, AND upregulated in Doxo vs. PBS AND concurrently downregulated in Doxo + ABT vs. Doxo). These lists included genes that were upregulated in the Doxo group, compared to the PBS group, and concurrently downregulated after ABT treatment (Figure 1, Tables S3 and S4). Genes showing this expression profile were considered high-priority candidates for being potential drivers of senescence. Genes from these lists were then examined for their role in biological processes based on published studies. Genes known to induce apoptosis upon silencing or inhibitor treatments were selected as potential undiscovered senolytic targets (Figure 2, Figure S4). We identified 10 such targets with this approach, four from FAPs and six from SCs (Figure 2, Table S5).

From the FAP analyses, we identified *Hmox1, Col1a1, Pltp,* and *Srxn1* (Figure 2, Table S5). These genes regulate apoptosis in various pathologies (Alaoui-Jamali et al., 2009; Dong et al., 2017; Liu et al., 2017; Zhou et al., 2015). *Hmox1* and *Srxn1* also have roles in cellular antioxidant defenses (Poss and Tonegawa, 1997; Zhou et al., 2015). From the SC analyses, we identified *Cryab, Rgcc, Rnf7, Fxyd3, Itga8,* and *Cav1* (Figure 2, Table S5), which also regulate apoptosis (Bibert et al., 2009; Chis et al., 2012; Duan et al., 1999; Kamradt et al., 2002; Khan et al., 2011; Percy et al., 2008; Sun and Li, 2013; Tirado et al., 2010; Wang et al., 2015; Xiao et al., 2017; Xu et al., 2014). *Cryab* and *Rnf7* also play roles in cellular antioxidant defenses (Fittipaldi et al., 2015; Sun and Li, 2013). Our next step was to validate these FAP and SC targets for expression upon senescence induction, and the potential to cause senolysis upon gene knockdown.

### CRYAB and HMOX1 are senolytic targets

To investigate the possibility of the 10 selected genes as senolytic targets upon knockdown, analyzed expression in primary mouse FAPs and SCs isolated by FACS (Figures 2, S5A, S5B, and S5C, Table S6) in conjunction with Doxo treatment. We determined that 250 or 100 nM Doxo induced senescence in FAPs and SCs. In FAPs, *Cryab*, *Hmox1*, *Pltp, Rnf7, Srxn1,* and *Rgcc* mRNAs significantly increased after Doxo treatment (Figure 2A); in SCs, mRNA levels of *Cryab*, *Hmox1,* and *Cav1* significantly increased (Figure 2A). Thus, *Cryab* and *Hmox1* appear to be common senolytic targets for both FAPs and SCs because of their robust increase in expression upon Doxo exposure.

Next, we suppressed *Cryab* or *Hmox1* expression using shRNA in cultured cells, and measured the viability of Doxo-treated and DMSO-treated FAPs (both non-senescent and senescent). We optimized the shRNAs for each gene by evaluating the efficacy of knockdown for up to five sequences specific for each gene. We further confirmed knockdown of these two genes by Western blot in FAPs and SCs (Figure 2B). *shRNA*s for *Cryab* or *Hmox1* significantly decreased cell viability 9 days after senescence induction (Figure 2C). In agreement with our viability results, *shRNAs* for each gene dramatically increased LDH release by senescent FAPs at Day 9 after senescence induction, indicating robust cell killing with our putative senolytic targets (Figure 2D). Similar results were not possible for SCs following transfection because of an inability to maintain SCs for prolonged periods in culture. LDH release by non-senescent FAP cells transfected with the *shRNA*s was not detected, indicating that senolysis was specific for senescent cells expressing the target genes.

### 25HC is a senolytic agent

**Figure 3:**
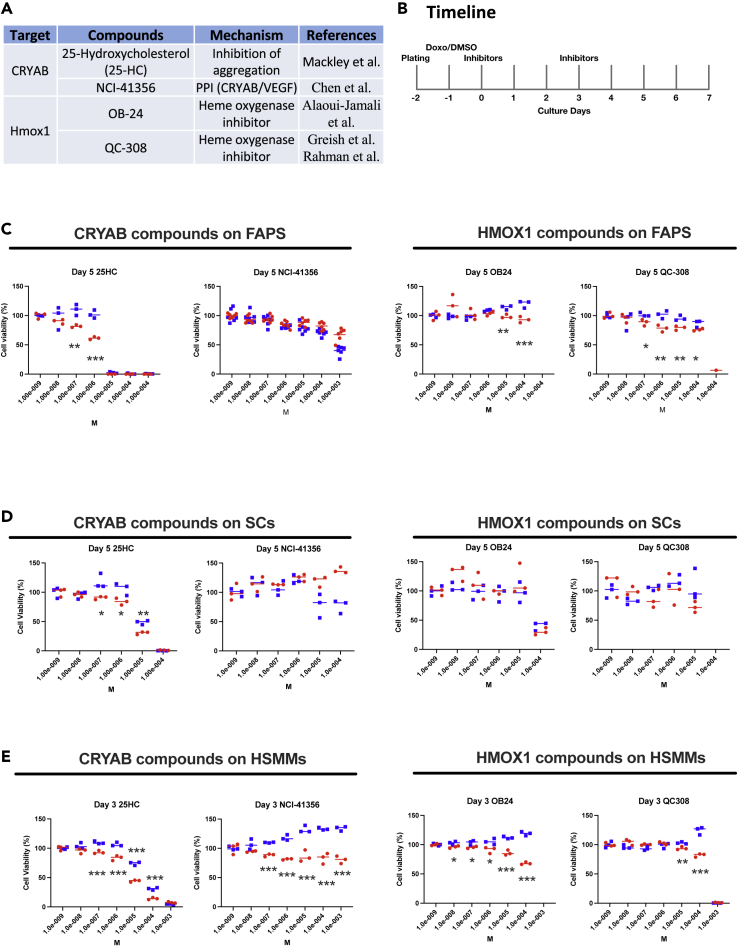
25HC is a senolytic in multiple muscle cell types (A) Targets, compounds, and mechanisms. (B) Timeline of cell viability assay with inhibitors. Cells were pre-cultured for 24 h, treated with Doxo for 24 h, and then treated with inhibitors. Cell viability was analyzed from day 0–7. (C–E) Effects of inhibitors on viability of FAPs, SCs, and HSMMs. Cells were treated with inhibitors at the indicated concentrations. Cell viability was analyzed on day 3 (HSMMs) or 5 (FAPs and SCs). Blue and red dots indicate non-senescent (NS) and senescent (SEN), respectively. The average value at 0 μM for each group was set at 100% cell viability. p values versus NS were obtained using unpaired two-tailed Student’s t test. ∗p < 0.05, ∗∗p < 0.01, and ∗∗∗p < 0.001.

We next examined the effects of chemical inhibitors of CRYAB (Chen et al., 2014; Makley et al., 2015) or HMOX1(Alaoui-Jamali et al., 2009; Greish et al., 2018; Rahman et al., 2012) for senolytic potential. We determined cell viability after exposing senescent and non-senescent cells to commercially available inhibitors for these targets (Figure 3A). We identified several small-molecule inhibitors of CRYAB and HMOX1 that were efficacious at killing senescent cells with minimal toxicity to non-senescent cells. The inhibitors and ABT were added to the media of cells 24 h after Doxo treatment (Figure 3B).

25HC specifically decreased the viability of senescent FAPs at concentrations between 0.1 and 1 mM, whereas NCI-41356 showed no difference in killing senescent versus non-senescent. In addition, OB24 and QC-308 slightly but significantly decreased the viability of FAPs in a senescence-dependent manner (Figure 3C).

**Figure 4:**
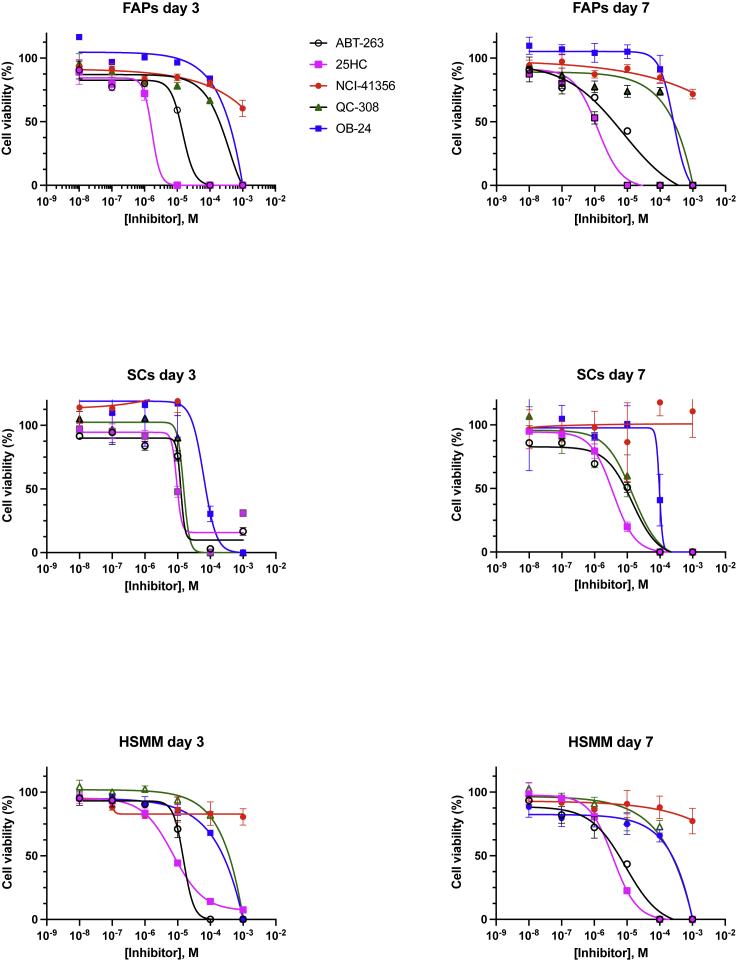
EC50 fitted curves (triplicates) of inhibitor effects at 3 and 7 days in FAPS and SCs EC50 was defined as the concentration of inhibitors that provokes a response halfway between baseline and maximum senolytic activity. EC50 values were determined by four- parameter logistic modeling using GraphPad Prism 8.0.

25HC also decreased the viability of senescent SCs, and OB24 and QC-308 showed no difference in killing (Figure 3D). As further validation for the senolytic potential of CRYAB and HMOX1 knockdown, we investigated the effects of these inhibitors on human skeletal muscle myoblasts (HSMMs), in which *CRYAB* and *HMOX1* were induced by Doxo in conjunction with other senescent marker genes (Figures S6A and S6B). These results showed that 25HC, OB24, and QC-308 significantly decreased cell viability, consistent with targeting senescent cells (Figure 3E). Further, calculating IC50’|'s for each of these candidate senolytics in FAPs, SCs, and HSMMs, showed that 25HC induced senolysis to levels similar or better to our positive control ABT (Figure 4, Table S7). Collectively, these data suggest that 25HC is a broad acting senolytic, independent of cell type or species.

### 25HC induces senolysis of mouse dermal fibroblasts (mDFs) and primary human cells from lung, heart, liver, kidney, and articular cartilage

**Figure 5:**
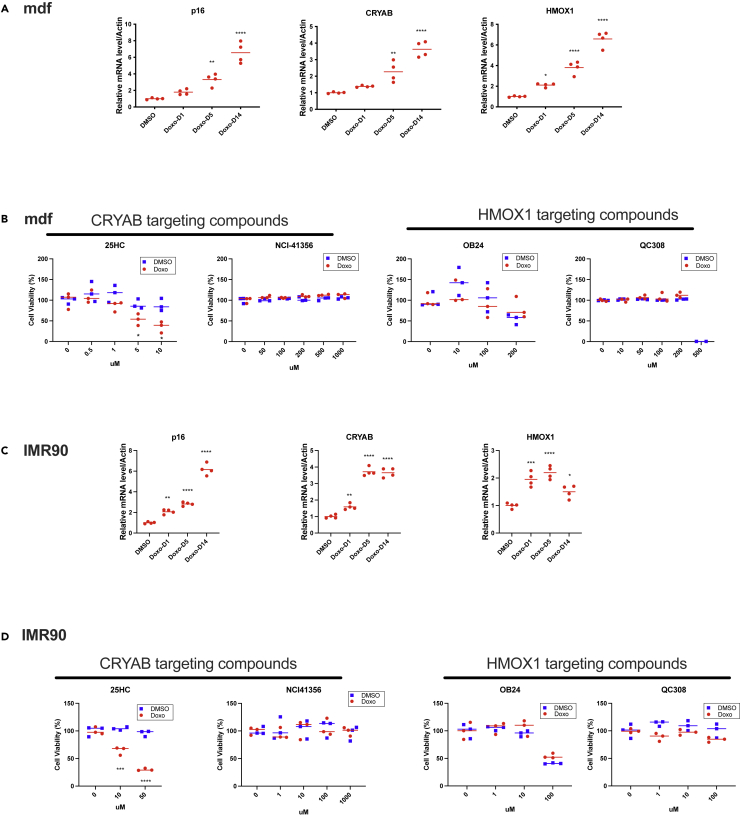
25HC induces senolysis in mDFs and IMR-90 cells (A and C) Doxo induced senescence marker *Cdkn2a* and senolytic targets *Cryab* and *Hmox1* mRNAs in (A) mDFs, and (C) IMR-90s. mDFs and IMR-90s were treated with 250 nM Doxo for 24 h. qRT-PCR was used to analyze RNA from DMSO-treated non-senescent (NS) mDFs and IMR-90s or Doxo-treated senescent (SEN) mDFs and IMR-90s at the indicated times after Doxo treatment. mRNA levels were normalized to the level of *actin* mRNA for DMSO- and Doxo-treated mDFs and IMR-90s. The average value of DMSO was set at 1. (B and D) Effects of CRYAB and HMOX1 inhibitors on DMSO-treated NS or Doxo-treated SEN cells; (B) mDFs, (D) IMR-90s. Cells were treated with the inhibitors at the indicated concentrations. Cell viability was measured after 72 h. (C) IMR-90 qRT-PCR. For qPCR: mean +/- SE, n =4, ∗p< 0.05, ∗∗p< 0.01, ∗∗∗p*<*0.001, ∗∗∗∗p*<*0.0001 by Dunnett’s multiple comparisons test vs DMSO. For cell viability assays: mean +/- SE, n =3, ∗∗p< 0.01, ∗∗∗p*<*0.001, ∗∗∗∗p*<*0.0001 vs 0 mM by student’s t test.

To determine whether CRYAB and HMOX1 inhibitors induce senolysis of cells other than those derived from skeletal muscle, we first tested mouse dermal fibroblasts (mDFs) and human lung fibroblasts (IMR-90). We treated both with DMSO (vehicle) or Doxo (250 nM) for 24 h to induce senescence (Figure 5 and S8A). mRNA analyses showed significant upregulation of the senescence marker *Cdkn2A* as well as CryAB and Hmox1 in senescent, but not non-senescent, cells (Figure 5). We next examined the effects of target inhibitors (Figure 5A) on both cell types (Figures 5B–5D). 25HC induced significant senolysis in mDF cells at five or 10 μM after 72 h of treatment, whereas treatment with all other compounds (i.e., NCI-41356, OB24, and QC308) was ineffective (Figure 5B). Likewise, in IMR-90 cells, 25HC induced significant senolysis (10 or 50 μM), but the other compounds had no effect (Figure 5D). Considering the results in Figures 4 and 5, we conclude that 25HC is an effective senolytic in multiple cell types of both mouse and human origin.

**Figure 6:**
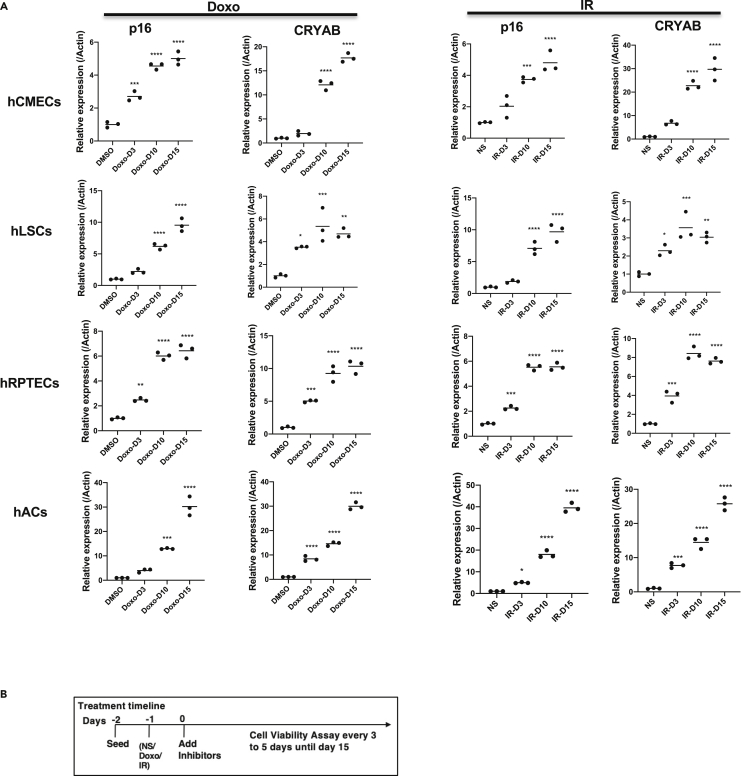
25HC induces senolysis in Doxo- and IR-induced senescent hCMECs, hLSCs, hRPTECs and hACs (A) Doxo and IR induced *Cdkn2a* and CryAB mRNAs in hCMECs, hLSCs, hRPTECs and hACs. Senescence was induced by treatment with 250 nM Doxo for 24 h or 10-Gy X-rays on all cell types. qRT-PCR was performed on RNA samples from DMSO/Doxo-treated or NS/IR treated cells at the indicated times. mRNA levels were normalized to actin mRNA. The average value of DMSO or NS was set at 1. For qRT-PCR: mean ± SE, n =4, ∗p< 0.05, ∗∗p< 0.01, ∗∗∗p*<*0.001, ∗∗∗∗p*<*0.0001 by Dunnett’s multiple comparisons test vs DMSO or NS, For cell viability assays: mean ± SE, n =3, ∗∗p< 0.01, ∗∗∗p*<*0.001, ∗∗∗∗p*<*0.0001 vs 0 mM by student’s t test.

**Figure 7:**
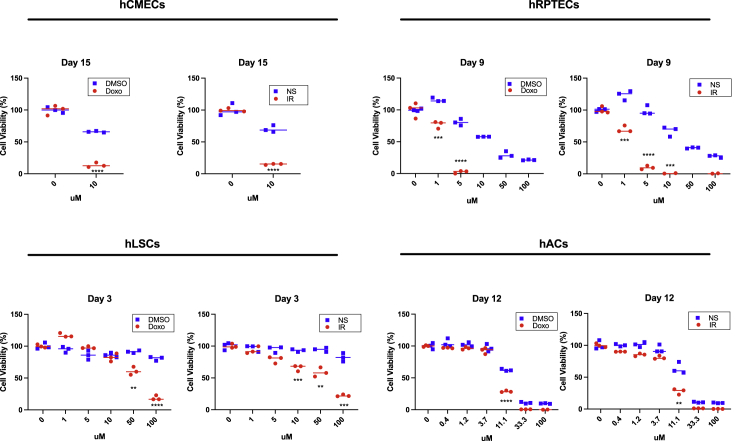
25HC treatment timeline and its effects on NS and SEN hCMECs, hLSCs, hRPTECs, and hACs All cell types were treated with either DMSO, 250 nM Doxo or 10-Gy IR at the times shown. 25HC treatment was started 24 h after Doxo or IR treatment. Cell viability was measured every 3–5 days for 15 days. Optimal treatment times for maximum senolysis are shown for each cell type: day 15 for hCMECs, day 3 for hLSCs, day 9 for hRPTECs and day 12 for hACs. For qRT-PCR: mean ± SE, n =4, ∗p< 0.05, ∗∗p< 0.01, ∗∗∗p*<*0.001, ∗∗∗∗p*<*0.0001 by Dunnett’s multiple comparisons test vs DMSO or NS, For cell viability assays: mean ± SE, n =3, ∗∗p< 0.01, ∗∗∗p*<*0.001, ∗∗∗∗p*<*0.0001 vs 0 mM by student’s t test.

We next evaluated the senolytic potential of 25HC for other primary human cells. Because senescence has been observed in several pathologies; of the heart (Katsuumi et al., 2018; Qi et al., 2015; Wang et al., 2016), liver (Nishizawa et al., 2016; Panebianco et al., 2017), kidney (Knoppert et al., 2019; Qi and Yang, 2018), and articular cartilage (Jeon et al., 2018; Loeser, 2009), we examined the senolytic potential of 25HC on human cardiac microvascular endothelial cells (hCMECs), human liver stellate cells (hLSCs), human renal proximal tubule epithelial cells (hRPTECs), and human articular chondrocytes (hAC). To induce senescence in these cells, we used either Doxo (250 nM) or X-irradiation (IR) (10 Gy) (Figure 6 and S8A). Senescence was confirmed by increased mRNA levels of *Cdkn2A*, *Lmnb1* mRNA decline, and the majority of cells were positive for SA-β-gal compared to non-senescent (NS) cells (Figures 6A, S8B, S9A, and S9B). All four cell types increased CryAB expression after Doxo or IR treatment, compared to NS samples (Figure 6A). Next, we evaluated the four cell types for senolysis in response to 25HC (Figure 6B). 25HC induced significant senolysis in hCMECs (10 μM after 15 days of treatment), hLSCs (100 μM after 3 days), hRPTECs (5 μM after 9 days), and hACs (11.1 μM after 12 days) (Figure 7). Other durations of 25HC treatment after inducing senescence with are shown in Figures S10–S13). Thus, 25HC is a senolytic agent for multiple mouse and human cell types. Overall, we demonstrated conservation of senolytic outcomes in two species and nine different cell types. Focusing on FAPs, we showed this cell type to be more sensitive to the senolytic effects of 25HC than the other cell types we tested.

### 25HC induces senolysis *in vivo* in doxorubicin-treated mice

**Figure 8:**
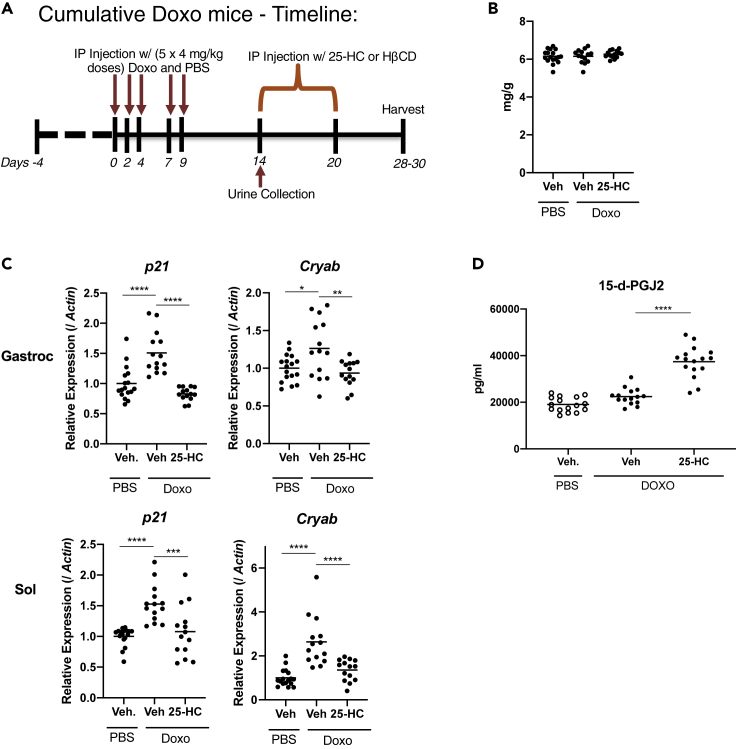
25HC induces senolysis *in vivo* in Doxo-treated mice (A) Timeline of 25HC treatment in Doxo-treated mice. Mice were given 4 mg/kg Doxo or PBS for 5 daily injections[A1], then injected with 25HC or vehicle for 7 consecutive days. (B) Mass of gastrocnemius muscles. Muscle weight was normalized to body weight. (C) *Cdkn2a* and *Cryab* mRNA levels in gastrocnemius (Gastroc) and soleus (Sol) muscles. RNA was collected 28–30 d after the first treatment with Doxo. mRNA levels were quantified by qRT-PCR, and normalized to *actin* mRNA levels. The average value of PBS-vehicle was set at 1. (D) 15-d-PGJ2 analysis in urine. Urine was collected at 24 h after the first treatment with 25HC or HbCD. p values versus PBS-Veh or Doxo-Veh are obtained using Tukey's multiple comparisons test. ∗p < 0.05, ∗∗p < 0.01, p < 0.001, and ∗∗∗∗p < 0.0001.

Having demonstrated the broad utility of 25HC in multiple cell types and two species in cells in culture, we asked whether 25HC was senolytic *in vivo*. We examined the effects of 25HC on Doxo-induced senescent cells *in vivo* in mice. We treated mice with PBS or 4 mg/kg Doxo (daily i.p. injection for 5 days), then administered 25HC or vehicle (HβCD) (Figure 8A). Doxo significantly decreased body weight, in agreement with prior studies (Gilliam et al., 2016; von Grabowiecki et al., 2015), with no difference in gastrocnemius muscle weight when normalized to body weight (Figure 8B). To confirm increased senescence of cells within muscle, we performed bulk quantitative mRNA analyses of senescent marker genes in gastrocnemius and soleus muscles. *Cdkn1A* mRNA significantly increased after Doxo treatment; however, *Cdkn2A* mRNA was not detected in either muscle. Consistent with our prior data showing efficacious killing of senescent cells in diverse cell types and species, 25HC significantly decreased *Cdkn1a* mRNA in both gastrocnemius and soleus. 25HC treatment also reduced *Cryab* mRNA levels (Figure 8C).

### Detection of senolysis by the biomarker 15 days-PGJ2 in urine of 25HC treated mice

A biomarker of *in vivo* senolysis was recently reported (Wiley et al., 2021). This biomarker is an intracellular lipid that has the potential to detect senescent cell killing *in vivo* by assaying for its presence in blood or urine, and can therefore be used as a proxy for senolysis *in vivo*. This lipid is the prostaglandin 1a,1b-dihomo-15-deoxy-delta-12,14-prostaglandin J2 (15-*d*ay-pGJ2), appears to be specific to senescent cells, and can be detected in body fluids of mice undergoing senolysis (Wiley et al., 2021). We assayed 15-*d*ay-PGJ2 in the urine of 25HC-treated mice that were treated with Doxo. In accord with other senolytics recently reported (Wiley et al., 2021), 15-*d*ay-PGJ2 levels increased significantly in the urine of 25HC-treated mice (Figure 8D), demonstrating that 25HC has senolytic effects in Doxo-induced senescence *in vivo*, consistent with our molecular, cellular, and *in vivo* data described above.

### 25HC induces senolysis in old male and female mice

**Figure 9:**
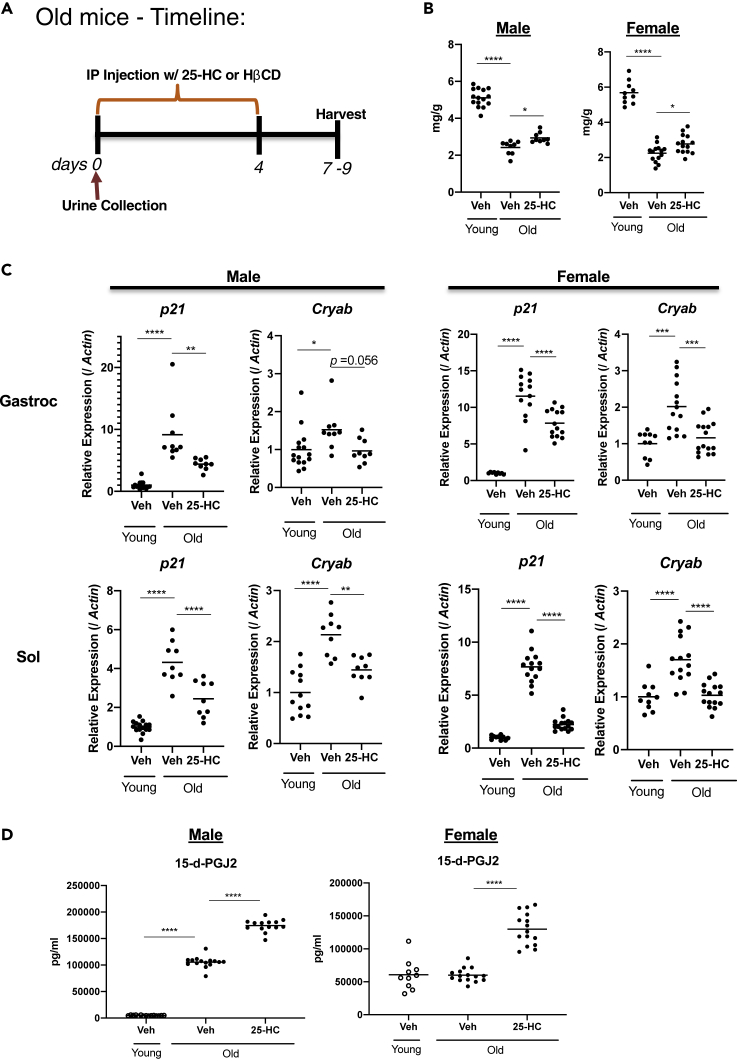
25HC is an in vivo senolytic in tissues of aged mice (A) Timeline of 25HC treatment in aged mice. Young (5 months old) and old (24–26 months old) mice were treated with 25HC or vehicle for 5 consecutive days. (B) Mass of gastrocnemius muscles in male and female mice. Muscle weight was normalized to body weight. (C) *Cdkn1a* and *Cryab* mRNA levels in gastrocnemius (Gastroc) and soleus (Sol) muscles. RNA was collected at 7–9 d after the first treatment with 25HC or vehicle. mRNA levels of the indicated genes were quantified by qRT-PCR, and normalized to those of *actin* mRNA. The average value of young-vehicle was set at 1. (D) 15-d-PGJ2 analysis in urine. Urine was collected 12 h after the first treatment with 25HC or vehicle. p values were obtained using Tukey's multiple comparisons test. ∗p< 0.05, ∗∗p< 0.01, ∗∗∗p< 0.001∗∗∗∗p< 0.0001.

To determine whether 25HC induces senolysis in naturally aged mice, we examined effects of 25HC on skeletal muscles of 24–26-month-old mice. Male and female mice were treated with 25HC or vehicle for 5 days (Figure 9A). Gastrocnemius muscle wet weights normalized to body weight were significantly decreased by aging, and 25HC restored ∼23% of muscle mass in both genders compared to vehicle-treated controls (Figure 9B). In agreement with the effects on muscle mass, 25HC significantly reduced *Cdkn1a* mRNA induction by aging in gastrocnemius and soleus muscles in both genders (Figure 9C). Strikingly, the level of the senolytic biomarker 15-*d*ay-PGJ2 was greater in the urine of 25HC-treated mice in both genders relative to controls. There also was a somewhat higher basal level of 15-*d*ay-PGJ2 in aged male mice compared to young controls (Figure 9D), suggesting a natural level of senolysis with age, potentially mediated by an immune process, which appeared to vary from animal to animal.

## Discussion

Our findings suggest that CRYAB is a gene to target for senolysis, and 25HC is a useful tool compound to develop senolytic drugs. CRYAB is upregulated upon senescence in nine different cell types and two species, suggesting it can be a common senolytic target for diseases of multiple organs. We speculate that CRYAB-mediated aging pathology may be because of cellular senescence resulting in higher expression of CRYAB. Therefore, targeting senescent cells via senolytics such as 25HC may lead to treatments for aging pathologies.

We showed broad utility for the senolytic potential of 25HC in various cell types, with the most robust outcomes in FAPs derived from skeletal muscle. 25HC is an oxysterol, an oxygenated derivative of cholesterol (Kloudova et al., 2017). In addition to blocking CRYAB aggregation (Makley et al., 2015), 25HC has been tested as an antiviral drug (Li et al., 2017; Liu et al., 2013). Notably, 25HC increases the inflammatory response, which leads to tissue damage in mice after influenza infection (Gold et al., 2014). 25HC also inhibits SARS-CoV-2 replication by depleting membrane cholesterol (Wang et al., 2020; Zang et al., 2020; Zu et al., 2020). Further, the mRNA encoding the 25HC synthesizing enzyme CH25H increases upon senescence in the retina (Francis Rodier and Ferbeyre, 2018). Considering 25HC has multiple roles in immune response, including beneficial and harmful effects, it will be important to further test 25HC in mice for safety and efficacy as a senolytic *in vivo*. Other oxysterols, including 24HC and 27HC (Kloudova et al., 2017), may also exert senolytic effects.

25HC showed stronger effects in skeletal muscle tissue compared to other tissues. Aging is associated with chronically increased inflammatory cytokines, such as interleukin 6 (IL-6), which are known inducers of muscle atrophy. 25HC substantially inhibited the expression of several SASP factors, including IL6, in skeletal muscle (Figures S17 and S18). This observation suggests that 25HC may improve age-related muscle atrophy via senolytic effects in future studies.

We also identified another potential senolytic target, Heme oxygenase 1. HMOX1 belongs to the heme oxygenase family, which mediates the first step in heme catabolism and plays crucial roles in heme degradation (Araujo et al., 2012). We demonstrated that HMOX1 inhibition by shRNAs and inhibitors causes senolysis in culture; however, the effects *in vivo* have yet to be characterized. Further analysis using more potent inhibitors or genetic modulation is required to address this issue *in vivo*.

We speculate that oxysterols may be a promising new class of senolytics with potential to treat age-related and viral diseases, such as COVID-19. Recently, there have been significant efforts to identify new senolytic targets and develop senolytic drugs for multiple age-associated pathologies (Hickson et al., 2019; Justice et al., 2019; van Deursen, 2019; Yousefzadeh et al., 2018). scRNA seq methods coupled to senescent model systems are a powerful method for discovering new classes of senolytics and targets because of the relatively low number of senescent cells in tissues, and their inherently heterogeneous nature. Each senolytic may have different efficacies based on tissue specificity, potency, and safety. Senolytics are in early stage clinical trials for multiple diseases, such as diabetes, Alzheimer’s disease, osteoarthritis, eye diseases, and COVID-19 (Kirkland and Tchkonia, 2020). The apparent tissue specificity of 25HC *in vivo* may relate to differential tissue accessibility within the animal, or relate to additional dosing optimization. Functional consequences of 25HC treatment in aged animals are a clear area for additional investigation in future studies. We do not yet know the mechanism of action of 25HC, but it may involve specifically downregulating CRYAB which in turn causes a proteotoxic stress within the senescent cell resulting in cell death and release of 15-dPGJ2.

In summary, our findings identified two senolytic target genes, namely CRYAB and HMOX1, and a senolytic tool compound, 25HC. 25HC is effective at targeting and killing senescent cells in both human and mouse cells from multiple tissues. As a class of senolytics, 25HC may lead to treatment strategies for senescence associated pathologies to improve health span.

### Limitations of the study

This study has several limitations. We determined broad spectrum senolytic action of 25HC in cultured cells from two species (human and mouse), and nine different cell types. However, we only verified *in vivo* activity of senolysis indirectly using the senolytic biomarker 15-dPGJ2. Discovery of robust gene expression or protein markers of senescent cells in multiple tissues would be helpful to validate a reduction in senescent cell burden as a result of treatment with 25HC. We also measured a small but significant increase in muscle wet weight of aged animals after treatment with 25HC. We have yet to ascertain functional improvement *in vivo* as a result of treatment with 25HC, and it will be interesting to determine if 25HC treatment improves other tissues or organ function in aged animals by reducing senescent cell burden.

## STAR★Methods

### Key resources table

**Table:** 

REAGENT or RESOURCE	SOURCE	IDENTIFIER
**Antibodies**		

anti-CD31	BioLegend	102514
anti-CD45	Biolegend	103122
anti-Sca-1	Invitrogen	12-5981-82
anti-PDGFRa	R&D	FAB1062P
anti-VCAM1	BioLegend	105720
anti-Integrin α7	MBL Lifesciences	K0046-4
anti-PDGFRa	Thermofisher	13-1401-82
anti-CRYAB	Santa Cruz Biotechnology	sc-137129
anti-HMOX1	Cell signaling Technology	82206

**Critical commercial assays**		

15-deoxy-D^12,14^-PGJ2 ELISA kit	Enzo Life Sciences	ADI-900-023

**Deposited data**		

Single Cell Sequencing data	GEO	GSE169531

**Experimental models: Organisms/strains**		

C57BL/6J mice	Jackson labs	000664

**Oligonucleotides**		

see Table S8		

### Resource availability

#### Lead contact

Further information and requests for resources and reagents should be directed to and will be fulfilled by the Lead Contact, Simon Melov (smelov@buckinstitute.org).

#### Materials availability

This study did not generate new unique reagents.

### Experimental model and subject details

#### *In-vivo* studies

All mice were maintained according to National Institutes of Health guidelines for use of live animals. All experimental procedures were approved by the Institutional Animal Care and Use Committee at the Buck Institute. Gender, genetic background and age of mice are described for specific experiments below.

#### *In-vivo* single-cell RNA seq experiments

Four-month-old male C57BL/6 wildtype (WT) mice were purchased from the Jackson Laboratory, and injected once intraperitoneally (i.p.) with 10 mg/kg doxorubicin hydrochloride (Doxo) (Tocris Bioscience, Product# 2252) in endotoxin-free Dulbecco′s PBS (w/o Ca^++^ and Mg^++^) (ETF-PBS) (EMD Millipore, Product TMS-012-A) or only ETF-PBS. After 6 days, the mice were treated with ABT263 in 10% ethanol, 30% PEG400, 60% Phosal 50 PG (vehicle) or vehicle only (Chang et al., 2016). ABT263 or vehicle was administered by gavage at 50 mg/kg/day for 5 consecutive days per cycle for 2 cycles with 9 days between cycles. At 14 days after the end of the 2^nd^ ABT263 or vehicle treatment cycle, the mice were euthanized and skeletal muscles were harvested.

#### *In-vivo* study of effects of 25HC on Doxo-treated mice

Male C57BL/6J mice (3–4 months old) were purchased from the Jackson Laboratory. We performed five *i.p.* injections with 4 mg/kg of Doxo (Tocris Bioscience, Product# 2252) for each injection over 10 days (Figure 6A). Therefore, we injected a total of 20 mg/kg of Doxo/mouse over 10 days. We used EFT-PBS (EMD Millipore, Product# TMS-012-A) as a control. To test effects of 25HC (Tocris Bioscience, Product# 5741) on Doxo-treated mice, we prepared three experimental groups (n=15 for each group): 1) PBS+HβCD vehicle (22.5%), 2) Doxo (20 mg/kg)+vehicle (22.5%), 3) Doxo (20 mg/kg)+25HC (50 mg/kg) (Figure 6A); 25HC was dissolved in 22.5% 2-hydroxypropyl-beta-cyclodextrin (HβCD) (Tocris Bioscience, Product 0708). On the Day 5 after the last Doxo/PBS injections, the mice were treated with either 25HC (50 mg/kg) or vehicle by *i.p.* injections for 7 consecutive days (Figure 6A). 12 hr after the first 25HC or vehicle treatments, urine was collected (Figure 6A) from each group to test for the senolysis marker 15-d-PGJ2. 1 wk after the last day of 25HC or vehicle treatments, the mice were euthanized over the period of 3 days (Figure 6A), and tissues were harvested for gene expression analysis. Senescence markers p16^INK4a^, p21^Cip1^ and CRYAB expression was analyzed on gastrocnemius (Gastroc), soleus (So), tibialis anterior (TA), visceral fat (VF), kidney (Ki), liver (Li), lung (Lu), heart (He), and skin (Sk) samples by qRT-PCR.

#### *In-vivo* testing of 25HC on aged mice

Two groups of male and female C57BL/6J mice were used. The young group was 5 months old, and the aged group was 24–26 months old. Six groups were prepared, 3 for male (n=9–15 mice) and 3 for female (n=10-16 mice/group) mice: 1) Young (5 months) + vehicle, 2) Old (24–26 months) + vehicle, 3) Old (24–26 months) + 25HC (Figure 7A). Each group was treated with either 25HC (50 mg/kg) or vehicle via *i.p.* injections for 5 consecutive days. After 12 h of the first treatments, urine samples were collected in low protein binding tubes (Figure 7A) to test for the senolysis marker 15-d-PGJ2. Mice were euthanized 2 days after the last injections over a period of 3 days (Figure 7A), and tissues were harvested for gene expression analysis. Senescence markers and CRYAB were analyzed using mRNA from gastrocnemius (Gastroc), soleus (So), tibialis anterior (TA), visceral fat (VF), kidney (Ki), liver (Li), lung (Lu), heart (He), and skin (Sk) samples using qRT-PCR (Figures S14, S15, S16, S17, and S18).

### Method details

#### 15-d-PGJ2 ELISA

Urine was collected at 12 or 24 h after the first treatment with 25HC or vehicle and diluted 10-fold with assay buffer. The amount of 15-d-PGJ2 was measured using a 15-deoxy-Δ^12,14^-PGJ2 ELISA kit according to manufactures’ protocol (Enzo Life Sciences).

#### Isolation of FAPs and SCs for single cell RNA-seq

Skeletal muscles were harvested from hind limbs of mice from PBS, Doxo or Doxo+ABT groups. Skeletal muscles from 5 mice were combined for each group. For skeletal muscle dissociation, a kit from Miltenyi Biotech was used (cat#130-098-305). Muscles were chopped into 2-4 mm pieces and enzymatic digestion was performed as described in the product datasheet using recommended kit enzymes, C Tubes, (Company: Miltenyi, Cat# 130-096-334), and gentle MACS Octo Dissociator with Heaters (cat# 130-096-427). After digestion, Debris Removal Solution (Company: Miltenyi, cat#130-109-398) was used to achieve cleaner single-cell suspensions. The suspension was stained with APC-CD31 (Biolegend, cat# 102514), APC-CD45 (Biolegend, cat# 103112), PB-Sca1(Invitrogen, cat# 12-5981-82), FITC-Integrin α7 (MBL life sciences, cat#K0046-4) and Biotin-PDGFRa(Thermo fisher, cat# 13-1401-82), PE/Cy7-Streptavidin (Thermo fisher, cat# SA1012) for FACS (FACS Aria II, BD Biosciences). FAPs were identified as Sca1+, PDGFra+, CD31-, CD45- and a7-integrin- and SCs were identified as a7-integrin+, Sca1-, PDGFra-, CD31-, and CD45- (Joe et al., 2010; Liu et al., 2015).

#### FAPs and satellite cells for primary culture

Hind limb muscles were digested in DMEM containing 0.5% (w/v) collagenase type II (Worthington) for 90 min at 37 °C with trituration and passed through a 40-μm nylon mesh. Erythrocytes were eliminated by treating with BD Pharm Lyse (BD Biosciences). Cells were resuspended in a washing buffer of PBS with 2% FBS and stained with antibodies for 30 min at 4 °C as described (Kamizaki et al., 2017). FAPs and SCs were isolated from the suspension by FACS (Aria II, BD Biosciences) using anti-CD31, CD45, Sca-1, PDGFR and VCAM1 antibodies. Antibodies are listed in Table S6. FAPs were identified as Sca1/PDGFR-positive and CD31-, CD45- and VCAM1-negative cells (Uezumi et al., 2010). SCs were identified as VCAM1-positive and CD31-, CD45-, Sca-1 and PDGFR-negative cells (Liu et al., 2015).

#### mDFs

For skin sample collection, mice were euthanized, hair removed from dorsal skin and the area cleaned with alcohol. A small 1 cm x 1 cm region of skin was removed, and subcutaneous fat removed by scalpel. Skin was digested in 2–3 ml of 0.25% trypsin at 37º C for 1 h. Hair and epidermis were further removed, and the dermis was minced into 2–3 mm pieces. Digestion of the sample in 5 ml of 1 mg/ml collagenase (Sigma, Cat#C5138) in HBSS (with Ca^++^ and Mg^++^) (Gibco, cat#24020117) was carried out at 37^0^ C on a shaker for 1 h. Collagenase digestion was stopped by addition of 10 ml 10% FBS DMEM. Filtration of the homogenate through a 70-micron mesh was performed to remove undigested tissue. Differential centrifugation and washing were performed at 1200 rpm for 5 min, and the pellet was resuspended in 10% FBS DMEM then seeded in a six-well plate. Media were changed at 5 days after seeding and then every 3 days.

#### 10X single-cell RNA-seq library prep

FAPs and SCs were isolated by FACS. Samples were prepared for 10X single-cell RNAseq to target 10 K cells. The 3′ single-cell library preparation was performed by following the 10X kit protocol. QC for cDNA and libraries were performed using Tapestation, according to the 10X protocol. Sequencing of the resultant libraries was then performed on Illumina sequencers as per the manufacturer’s protocols.

#### 10X single-cell RNA-seq analysis

Libraries were processed through CellRanger v2.1.0 [1], aligned to mouse genome version mm10. Data analysis was performed with Seurat version 2.3(Stuart et al., 2019) by integrating libraries from control, DOXO treated, and DOXO+ABT263 treated animals. Differential gene expression analysis across clusters was performed between integrated library pairs (Doxo vs. PBS, Doxo+ABT vs Doxo). Clusters with significant (p < 0.05) expression changes in senescence marker genes between treatment groups were further analyzed.

#### Cell culture

All experiments were performed in incubators at 37 °C, 3% O_2_ and 5% CO_2_. Senescence was induced by treatment with either Doxo (250 nM for 24 h) (Demaria et al., 2017) or X-irradiation (10 Gy) (Limbad et al., 2020). For cell culture studies using 25HC, it was used at the indicated concentrations in the media. 25HC was dissolved in 100% EtOH, such that the total concentration of EtOH in the media did not exceed 0.5%, and vehicle controls used were equivalent volumes of EtOH without 25HC.

#### FAP cells, SCs, and HSMM

FAPs and SCs were cultured on Matrigel-coated dishes (BD Biosciences) in growth medium (GM) consisting of DMEM plus 10% FBS, 1% penicillin-streptomycin and 10% MyoCult (STEMCELL Technologies) for 7 days. Human myogenic cell HSMMs (Lonza) were maintained according to the manufacturer’s instructions with SkGM-2 medium (Lonza).

#### Human cells

IMR-90 cells were cultured in DMEM with 10% FBS. Cardiac endothelial cells (Sciencell, Catalog 6000, Lot 15367), liver stellate cells (Sciencell, Catalog 5300, Lot 25907), renal proximal tubule epithelial cells (Sciencell, Catalog 4100, Lot 19870) and articular chondrocytes (Sciencell, Catalog 4650, Lot 69440) were cultured in media from Sciencell as per the datasheets.

#### Quantitative RT-PCR (qRT-PCR)

Total RNA was extracted using either Isolate II RNA mini kit (Bio-52073) or RNeasy Mini Kit (Qiagen), then reverse transcribed using PrimeScript RT Reagent Kit (Perfect Real Time) (Takara Bio USA, Prod RR037B). qRT-PCR reactions were performed using TB Green Premix Ex Taq (Tli RNase H Plus) (Takara Bio USA, Prod RR420L). The amounts of mRNA were normalized relative to those of *Actin* mRNA. Specific primer sequences used for qRT-PCR are listed in Table S8.

#### shRNA

Experiments were performed in triplicate. Cells were seeded (2000 cells/well) in 96-well plates. 24 h later, cells were infected with either a scrambled sequence (control), shCRYAB or shHMOX1 lentiviruses at 5 MOI in culture medium with 0.1% polybrene. Control and shRNAs were from Mission SHRNA Custom Lentiviral Particles. Each vial contained 10^6^ TU/mL. For HMOX1, TRCN0000218100 TRC2 and TRCN0000234075 TRC2 were used. For CRYAB, TRCN0000097213 TRC1 and TRCN0000097210 TRC1 were used. SHC202V TRC2 and SHC002V TRC1 non-targeting controls were used. 18 h after infection, virus was removed and fresh media added for 48–72 h. Puromycin (2 μg/mL) selection was performed for 3 days, after which knockdown (KD) of the targeted gene was confirmed by qRT-PCR and Westerns, and cells were treated with Doxo (250 nM) for 24 h to induce senescence. Cell viability was assessed every 2–3 days to determine senolysis.

#### Cell viability assay

Cell viability was assessed using a cell counting kit-8 from Dojindo Molecular Technologies, Inc (Cat CK04-20). Cells were incubated with culture media + 10% kit cell viability reagent, 150 μL/well in 96-well plates for 2 h. The supernatant was transferred to a new 96-well plate and absorbance read at 450 nm as recommended in the product datasheet.

#### Cell death assay

Cell death was analyzed by the Cytotoxicity LDH Assay Kit (Dojindo), according to manufacturer’s protocol. Briefly, the supernatant was collected 7 d after removing medium containing Doxo, mixed with the same volume of working solution, incubated for 30 min at 37 °C in a humidified 5% CO_2_/3% O_2_ atmosphere, and then absorbance at 490 nm was measured with a microplate reader.

#### SA-β-gal assay

SA-β-gal staining was performed using the Biovision kit (Prod# K320-250). Cells were plated at 5K/cm^2^. SA-β-gal was assessed 24–48 h later, when cells were 60%–70% confluent. Cells were washed with PBS and fixed for 5 min. After another PBS wash, cells were incubated overnight at 37º C in staining solution. After washing with PBS, a minimum of 300 cells were counted. Positive (blue) cells were scored as percentage of total cell number.

### Quantification and statistical analysis

Statistical analyses were conducted using unpaired two-tailed Student’s t-test and Dunnett's multiple comparison test using GraphPad Prism 9 software (GraphPad Software, Inc.). Statistical significance was assigned for *p*-values <0.05.

## Data Availability

Single-cell RNA-seq counts and raw data have been posted on Gene Expression Omnibus (GEO), and is publicly available at the time of publication. Accession number is listed in the key resources table.

## Supplemental information

Document S1. Figures S1–S18 and Tables S1, S2, and S5–S8

Table S3. FAPs – List of differentially expressed genes from either p16 or p21 expressing clusters after applying following filters p value of <0.05, average logFC <= -0.25 or >=0.25, and upregulated in Doxo vs. PBS and concurrently downregulated in Doxo+ABT vs. Doxo.

Table S4. SCs –List of differentially expressed genes from either p16 or p21 expressing clusters after applying following filters p value of <0.05, average logFC <= -0.25 or >=0.25, and upregulated in Doxo vs. PBS and concurrently downregulated in Doxo+ABT vs. Doxo.

## References

[bib1] Aguayo-Mazzucato C., Andle J., Lee T.B., Midha A., Talemal L., Chipashvili V., Hollister-Lock J., van Deursen J., Weir G., Bonner-Weir S. (2019). Acceleration of beta cell aging determines diabetes and senolysis improves disease outcomes. Cell Metab..

[bib2] Alaoui-Jamali M.A., Bismar T.A., Gupta A., Szarek W.A., Su J., Song W., Xu Y., Xu B., Liu G., Vlahakis J.Z. (2009). A novel experimental heme oxygenase-1-targeted therapy for hormone-refractory prostate cancer. Cancer Res..

[bib3] Anderson R., Lagnado A., Maggiorani D., Walaszczyk A., Dookun E., Chapman J., Birch J., Salmonowicz H., Ogrodnik M., Jurk D. (2019). Length-independent telomere damage drives post-mitotic cardiomyocyte senescence. EMBO J..

[bib4] Araujo J.A., Zhang M., Yin F. (2012). Heme oxygenase-1, oxidation, inflammation, and atherosclerosis. Front. Pharmacol..

[bib5] Bernadotte A., Mikhelson V.M., Spivak I.M. (2016). Markers of cellular senescence. Telomere shortening as a marker of cellular senescence. Aging (Albany NY).

[bib6] Bibert S., Aebischer D., Desgranges F., Roy S., Schaer D., Kharoubi-Hess S., Horisberger J.D., Geering K. (2009). A link between FXYD3 (Mat-8)-mediated Na,K-ATPase regulation and differentiation of Caco-2 intestinal epithelial cells. Mol. Biol. Cell.

[bib7] Brbic M., Zitnik M., Wang S., Pisco A.O., Altman R.B., Darmanis S., Leskovec J. (2020). MARS: discovering novel cell types across heterogeneous single-cell experiments. Nat. Methods.

[bib8] Bussian T.J., Aziz A., Meyer C.F., Swenson B.L., van Deursen J.M., Baker D.J. (2018). Clearance of senescent glial cells prevents tau-dependent pathology and cognitive decline. Nature.

[bib9] Carter R.A., Bihannic L., Rosencrance C., Hadley J.L., Tong Y., Phoenix T.N., Natarajan S., Easton J., Northcott P.A., Gawad C. (2018). A single-cell transcriptional atlas of the developing murine cerebellum. Curr. Biol..

[bib10] Chang J., Wang Y., Shao L., Laberge R.M., Demaria M., Campisi J., Janakiraman K., Sharpless N.E., Ding S., Feng W. (2016). Clearance of senescent cells by ABT263 rejuvenates aged hematopoietic stem cells in mice. Nat. Med..

[bib11] Chatterjee K., Zhang J., Honbo N., Karliner J.S. (2010). Doxorubicin cardiomyopathy. Cardiology.

[bib12] Chen Z., Ruan Q., Han S., Xi L., Jiang W., Jiang H., Ostrov D.A., Cai J. (2014). Discovery of structure-based small molecular inhibitor of alphaB-crystallin against basal-like/triple-negative breast cancer development in vitro and in vivo. Breast Cancer Res. Treat.

[bib13] Chiche A., Le Roux I., von Joest M., Sakai H., Aguin S.B., Cazin C., Salam R., Fiette L., Alegria O., Flamant P. (2017). Injury-induced senescence enables in vivo reprogramming in skeletal muscle. Cell Stem Cell.

[bib14] Chis R., Sharma P., Bousette N., Miyake T., Wilson A., Backx P.H., Gramolini A.O. (2012). alpha-Crystallin B prevents apoptosis after H2O2 exposure in mouse neonatal cardiomyocytes. Am. J. Physiol. Heart Circ. Physiol..

[bib15] Demaria M., O'Leary M.N., Chang J., Shao L., Liu S., Alimirah F., Koenig K., Le C., Mitin N., Deal A.M. (2017). Cellular senescence promotes adverse effects of chemotherapy and cancer relapse. Cancer Discov..

[bib16] Dong W., Gong H., Zhang G., Vuletic S., Albers J., Zhang J., Liang H., Sui Y., Zheng J. (2017). Lipoprotein lipase and phospholipid transfer protein overexpression in human glioma cells and their effect on cell growth, apoptosis, and migration. Acta Biochim. Biophys. Sin (Shanghai).

[bib17] Duan H., Wang Y., Aviram M., Swaroop M., Loo J.A., Bian J., Tian Y., Mueller T., Bisgaier C.L., Sun Y. (1999). SAG, a novel zinc RING finger protein that protects cells from apoptosis induced by redox agents. Mol. Cell Biol..

[bib18] Fittipaldi S., Mercatelli N., Dimauro I., Jackson M.J., Paronetto M.P., Caporossi D. (2015). Alpha B-crystallin induction in skeletal muscle cells under redox imbalance is mediated by a JNK-dependent regulatory mechanism. Free Radic. Biol. Med..

[bib19] Francis Rodier D.Z., Ferbeyre G. (2018). Cellular senescence, geroscience, cancer and beyond. Aging (Albany NY).

[bib20] Fu Y., Huang X., Zhang P., van de Leemput J., Han Z. (2020). Single-cell RNA sequencing identifies novel cell types in Drosophila blood. J. Genet. Genomics.

[bib21] Fuhrmann-Stroissnigg H., Ling Y.Y., Zhao J., McGowan S.J., Zhu Y., Brooks R.W., Grassi D., Gregg S.Q., Stripay J.L., Dorronsoro A. (2017). Identification of HSP90 inhibitors as a novel class of senolytics. Nat. Commun..

[bib22] Gilliam L.A., Lark D.S., Reese L.R., Torres M.J., Ryan T.E., Lin C.T., Cathey B.L., Neufer P.D. (2016). Targeted overexpression of mitochondrial catalase protects against cancer chemotherapy-induced skeletal muscle dysfunction. Am. J. Physiol. Endocrinol. Metab..

[bib23] Gilliam L.A., St Clair D.K. (2011). Chemotherapy-induced weakness and fatigue in skeletal muscle: the role of oxidative stress. Antioxid. Redox Signal..

[bib24] Gold E.S., Diercks A.H., Podolsky I., Podyminogin R.L., Askovich P.S., Treuting P.M., Aderem A. (2014). 25-Hydroxycholesterol acts as an amplifier of inflammatory signaling. Proc. Natl. Acad. Sci. U S A.

[bib25] Gonzalez-Gualda E., Paez-Ribes M., Lozano-Torres B., Macias D., Wilson J.R., Gonzalez-Lopez C., Ou H.L., Miron-Barroso S., Zhang Z., Lerida-Viso A. (2020). Galacto-conjugation of Navitoclax as an efficient strategy to increase senolytic specificity and reduce platelet toxicity. Aging Cell.

[bib26] Greish K.F., Salerno L., Al Zahrani R., Amata E., Modica M.N., Romeo G., Marrazzo A., Prezzavento O., Sorrenti V., Rescifina A. (2018). Novel structural insight into inhibitors of heme oxygenase-1 (HO-1) by new imidazole-based compounds: biochemical and in vitro anticancer activity evaluation. Molecules.

[bib27] Grun D., Lyubimova A., Kester L., Wiebrands K., Basak O., Sasaki N., Clevers H., van Oudenaarden A. (2015). Single-cell messenger RNA sequencing reveals rare intestinal cell types. Nature.

[bib28] Hickson L.J., Langhi Prata L.G.P., Bobart S.A., Evans T.K., Giorgadze N., Hashmi S.K., Herrmann S.M., Jensen M.D., Jia Q., Jordan K.L. (2019). Senolytics decrease senescent cells in humans: preliminary report from a clinical trial of Dasatinib plus Quercetin in individuals with diabetic kidney disease. EBioMedicine.

[bib29] Jeon O.H., David N., Campisi J., Elisseeff J.H. (2018). Senescent cells and osteoarthritis: a painful connection. J. Clin. Invest..

[bib30] Joe A.W., Yi L., Natarajan A., Le Grand F., So L., Wang J., Rudnicki M.A., Rossi F.M. (2010). Muscle injury activates resident fibro/adipogenic progenitors that facilitate myogenesis. Nat. Cell Biol..

[bib31] Justice J.N., Nambiar A.M., Tchkonia T., LeBrasseur N.K., Pascual R., Hashmi S.K., Prata L., Masternak M.M., Kritchevsky S.B., Musi N., Kirkland J.L. (2019). Senolytics in idiopathic pulmonary fibrosis: results from a first-in-human, open-label, pilot study. EBioMedicine.

[bib32] Kamizaki K., Doi R., Hayashi M., Saji T., Kanagawa M., Toda T., Fukada S.I., Ho H.H., Greenberg M.E., Endo M., Minami Y. (2017). The Ror1 receptor tyrosine kinase plays a critical role in regulating satellite cell proliferation during regeneration of injured muscle. J. Biol. Chem..

[bib33] Kamradt M.C., Chen F., Sam S., Cryns V.L. (2002). The small heat shock protein alpha B-crystallin negatively regulates apoptosis during myogenic differentiation by inhibiting caspase-3 activation. J. Biol. Chem..

[bib34] Katsuumi G., Shimizu I., Yoshida Y., Minamino T. (2018). Vascular senescence in cardiovascular and metabolic diseases. Front. Cardiovasc. Med..

[bib35] Keeney J.T.R., Ren X., Warrier G., Noel T., Powell D.K., Brelsfoard J.M., Sultana R., Saatman K.E., Clair D.K.S., Butterfield D.A. (2018). Doxorubicin-induced elevated oxidative stress and neurochemical alterations in brain and cognitive decline: protection by MESNA and insights into mechanisms of chemotherapy-induced cognitive impairment ("chemobrain"). Oncotarget.

[bib36] Khan S., Lakhe-Reddy S., McCarty J.H., Sorenson C.M., Sheibani N., Reichardt L.F., Kim J.H., Wang B., Sedor J.R., Schelling J.R. (2011). Mesangial cell integrin alphavbeta8 provides glomerular endothelial cell cytoprotection by sequestering TGF-beta and regulating PECAM-1. Am. J. Pathol..

[bib37] Kim E.C., Kim J.R. (2019). Senotherapeutics: emerging strategy for healthy aging and age-related disease. BMB Rep..

[bib38] Kirkland J.L., Tchkonia T. (2017). Cellular senescence: a translational perspective. EBioMedicine.

[bib39] Kirkland J.L., Tchkonia T. (2020). Senolytic drugs: from discovery to translation. J. Intern. Med..

[bib40] Kloudova A., Guengerich F.P., Soucek P. (2017). The role of oxysterols in human cancer. Trends Endocrinol. Metab..

[bib41] Knoppert S.N., Valentijn F.A., Nguyen T.Q., Goldschmeding R., Falke L.L. (2019). Cellular senescence and the kidney: potential therapeutic targets and tools. Front. Pharmacol..

[bib42] Li C., Deng Y.Q., Wang S., Ma F., Aliyari R., Huang X.Y., Zhang N.N., Watanabe M., Dong H.L., Liu P. (2017). 25-Hydroxycholesterol protects host against zika virus infection and its associated microcephaly in a mouse model. Immunity.

[bib43] Limbad C., Oron T.R., Alimirah F., Davalos A.R., Tracy T.E., Gan L., Desprez P.Y., Campisi J. (2020). Astrocyte senescence promotes glutamate toxicity in cortical neurons. PLoS One.

[bib44] Liu L., Cheung T.H., Charville G.W., Rando T.A. (2015). Isolation of skeletal muscle stem cells by fluorescence-activated cell sorting. Nat. Protoc..

[bib45] Liu S., Liao G., Li G. (2017). Regulatory effects of COL1A1 on apoptosis induced by radiation in cervical cancer cells. Cancer Cell Int..

[bib46] Liu S.Y., Aliyari R., Chikere K., Li G., Marsden M.D., Smith J.K., Pernet O., Guo H., Nusbaum R., Zack J.A. (2013). Interferon-inducible cholesterol-25-hydroxylase broadly inhibits viral entry by production of 25-hydroxycholesterol. Immunity.

[bib47] Loeser R.F. (2009). Aging and osteoarthritis: the role of chondrocyte senescence and aging changes in the cartilage matrix. Osteoarthritis Cartilage.

[bib48] Lukjanenko L., Karaz S., Stuelsatz P., Gurriaran-Rodriguez U., Michaud J., Dammone G., Sizzano F., Mashinchian O., Ancel S., Migliavacca E. (2019). Aging disrupts muscle stem cell function by impairing matricellular WISP1 secretion from fibro-adipogenic progenitors. Cell Stem Cell.

[bib49] Makley L.N., McMenimen K.A., DeVree B.T., Goldman J.W., McGlasson B.N., Rajagopal P., Dunyak B.M., McQuade T.J., Thompson A.D., Sunahara R. (2015). Pharmacological chaperone for alpha-crystallin partially restores transparency in cataract models. Science.

[bib50] Nishizawa H., Iguchi G., Fukuoka H., Takahashi M., Suda K., Bando H., Matsumoto R., Yoshida K., Odake Y., Ogawa W., Takahashi Y. (2016). IGF-I induces senescence of hepatic stellate cells and limits fibrosis in a p53-dependent manner. Sci. Rep..

[bib51] Panebianco C., Oben J.A., Vinciguerra M., Pazienza V. (2017). Senescence in hepatic stellate cells as a mechanism of liver fibrosis reversal: a putative synergy between retinoic acid and PPAR-gamma signalings. Clin. Exp. Med..

[bib52] Papalexi E., Satija R. (2018). Single-cell RNA sequencing to explore immune cell heterogeneity. Nat. Rev. Immunol..

[bib53] Percy C.J., Pat B.K., Healy H., Johnson D.W., Gobe G.C. (2008). Phosphorylation of caveolin-1 is anti-apoptotic and promotes cell attachment during oxidative stress of kidney cells. Pathology.

[bib54] Poss K.D., Tonegawa S. (1997). Reduced stress defense in heme oxygenase 1-deficient cells. Proc. Natl. Acad. Sci. U S A.

[bib55] Qi R., Yang C. (2018). Renal tubular epithelial cells: the neglected mediator of tubulointerstitial fibrosis after injury. Cell Death Dis..

[bib56] Qi X.F., Chen Z.Y., Xia J.B., Zheng L., Zhao H., Pi L.Q., Park K.S., Kim S.K., Lee K.J., Cai D.Q. (2015). FoxO3a suppresses the senescence of cardiac microvascular endothelial cells by regulating the ROS-mediated cell cycle. J. Mol. Cell Cardiol..

[bib57] Rahman M.N., Vlahakis J.Z., Vukomanovic D., Lee W., Szarek W.A., Nakatsu K., Jia Z. (2012). A novel, "double-clamp" binding mode for human heme oxygenase-1 inhibition. PLoS One.

[bib58] Rodier F., Campisi J. (2011). Four faces of cellular senescence. J. Cell Biol..

[bib59] Rubenstein A.B., Smith G.R., Raue U., Begue G., Minchev K., Ruf-Zamojski F., Nair V.D., Wang X., Zhou L., Zaslavsky E. (2020). Single-cell transcriptional profiles in human skeletal muscle. Sci. Rep..

[bib60] Saito Y., Chikenji T.S., Matsumura T., Nakano M., Fujimiya M. (2020). Exercise enhances skeletal muscle regeneration by promoting senescence in fibro-adipogenic progenitors. Nat. Commun..

[bib61] Sapieha P., Mallette F.A. (2018). Cellular senescence in postmitotic cells: beyond growth arrest. Trends Cell Biol..

[bib62] Skelly D.A., Squiers G.T., McLellan M.A., Bolisetty M.T., Robson P., Rosenthal N.A., Pinto A.R. (2018). Single-cell transcriptional profiling reveals cellular diversity and intercommunication in the mouse heart. Cell Rep..

[bib63] Sousa-Victor P., Gutarra S., Garcia-Prat L., Rodriguez-Ubreva J., Ortet L., Ruiz-Bonilla V., Jardi M., Ballestar E., Gonzalez S., Serrano A.L. (2014). Geriatric muscle stem cells switch reversible quiescence into senescence. Nature.

[bib64] Stuart T., Butler A., Hoffman P., Hafemeister C., Papalexi E., Mauck W.M., Hao Y., Stoeckius M., Smibert P., Satija R. (2019). Comprehensive integration of single-cell data. Cell.

[bib65] Sun Y., Li H. (2013). Functional characterization of SAG/RBX2/ROC2/RNF7, an antioxidant protein and an E3 ubiquitin ligase. Protein Cell.

[bib66] Tirado O.M., MacCarthy C.M., Fatima N., Villar J., Mateo-Lozano S., Notario V. (2010). Caveolin-1 promotes resistance to chemotherapy-induced apoptosis in Ewing's sarcoma cells by modulating PKCalpha phosphorylation. Int. J. Cancer.

[bib67] Uezumi A., Fukada S., Yamamoto N., Takeda S., Tsuchida K. (2010). Mesenchymal progenitors distinct from satellite cells contribute to ectopic fat cell formation in skeletal muscle. Nat. Cell Biol..

[bib68] van Deursen J.M. (2019). Senolytic therapies for healthy longevity. Science.

[bib69] van Norren K., van Helvoort A., Argiles J.M., van Tuijl S., Arts K., Gorselink M., Laviano A., Kegler D., Haagsman H.P., van der Beek E.M. (2009). Direct effects of doxorubicin on skeletal muscle contribute to fatigue. Br. J. Cancer.

[bib70] von Grabowiecki Y., Licona C., Palamiuc L., Abreu P., Vidimar V., Coowar D., Mellitzer G., Gaiddon C. (2015). Regulation of a Notch3-Hes1 pathway and protective effect by a tocopherol-omega alkanol chain derivative in muscle atrophy. J. Pharmacol. Exp. Ther..

[bib71] Walaszczyk A., Dookun E., Redgrave R., Tual-Chalot S., Victorelli S., Spyridopoulos I., Owens A., Arthur H.M., Passos J.F., Richardson G.D. (2019). Pharmacological clearance of senescent cells improves survival and recovery in aged mice following acute myocardial infarction. Aging Cell.

[bib72] Wang S., Li W., Hui H., Tiwari S.K., Zhang Q., Croker B.A., Rawlings S., Smith D., Carlin A.F., Rana T.M. (2020). Cholesterol 25-Hydroxylase inhibits SARS-CoV-2 and other coronaviruses by depleting membrane cholesterol. EMBO J..

[bib73] Wang W.W., Wang Y.B., Wang D.Q., Lin Z., Sun R.J. (2015). Integrin beta-8 (ITGB8) silencing reverses gefitinib resistance of human hepatic cancer HepG2/G cell line. Int. J. Clin. Exp. Med..

[bib74] Wang Y., Boerma M., Zhou D. (2016). Ionizing radiation-induced endothelial cell senescence and cardiovascular diseases. Radiat. Res..

[bib75] Wiley C.D., Sharma R., Davis S.S., Lopez-Dominguez J.A., Mitchell K.P., Wiley S., Alimirah F., Kim D.E., Payne T., Rosko A. (2021). Oxylipin biosynthesis reinforces cellular senescence and allows detection of senolysis. Cell Metab..

[bib76] Wissler Gerdes E.O., Zhu Y., Tchkonia T., Kirkland J.L. (2020). Discovery, development, and future application of senolytics: theories and predictions. FEBS J..

[bib77] Xiao Y., Jiang Y., Song H., Liang T., Li Y., Yan D., Fu Q., Li Z. (2017). RNF7 knockdown inhibits prostate cancer tumorigenesis by inactivation of ERK1/2 pathway. Sci. Rep..

[bib78] Xu M., Pirtskhalava T., Farr J.N., Weigand B.M., Palmer A.K., Weivoda M.M., Inman C.L., Ogrodnik M.B., Hachfeld C.M., Fraser D.G. (2018). Senolytics improve physical function and increase lifespan in old age. Nat. Med..

[bib79] Xu R., Shang C., Zhao J., Han Y., Liu J., Chen K., Shi W. (2014). Knockdown of response gene to complement 32 (RGC32) induces apoptosis and inhibits cell growth, migration, and invasion in human lung cancer cells. Mol. Cell Biochem..

[bib80] Yousefzadeh M.J., Zhu Y., McGowan S.J., Angelini L., Fuhrmann-Stroissnigg H., Xu M., Ling Y.Y., Melos K.I., Pirtskhalava T., Inman C.L. (2018). Fisetin is a senotherapeutic that extends health and lifespan. EBioMedicine.

[bib81] Zang R., Case J.B., Yutuc E., Ma X., Shen S., Gomez Castro M.F., Liu Z., Zeng Q., Zhao H., Son J. (2020). Cholesterol 25-hydroxylase suppresses SARS-CoV-2 replication by blocking membrane fusion. Proc. Natl. Acad. Sci. U S A.

[bib82] Zhou Y., Zhou Y., Yu S., Wu J., Chen Y., Zhao Y. (2015). Sulfiredoxin-1 exerts anti-apoptotic and neuroprotective effects against oxidative stress-induced injury in rat cortical astrocytes following exposure to oxygen-glucose deprivation and hydrogen peroxide. Int. J. Mol. Med..

[bib83] Zu S., Deng Y.Q., Zhou C., Li J., Li L., Chen Q., Li X.F., Zhao H., Gold S., He J. (2020). 25-Hydroxycholesterol is a potent SARS-CoV-2 inhibitor. Cell Res..

