# Supplementary material for: Senolysis induced by 25-hydroxycholesterol targets CRYAB in multiple cell types
Source: iScience. 2022 Feb 2;25(2):103848. doi: 10.1016/j.isci.2022.103848 (PMC8851282; doi:10.1016/j.isci.2022.103848)
Supplement: Document S1. Figures S1–S18 and Tables S1, S2, and S5–S8 [file mmc1.pdf]

iScience, Volume *25*

Supplemental information

Senolysis induced by 25-hydroxycholesterol

targets CRYAB in multiple cell types

Chandani Limbad, Ryosuke Doi, Julia McGirr, Serban Ciotlos, Kevin Perez, Zachary S. Clayton, Radha Daya, Douglas R. Seals, Judith Campisi, and Simon Melov

Supplementary Material
Limbad et al. 2022

(a)

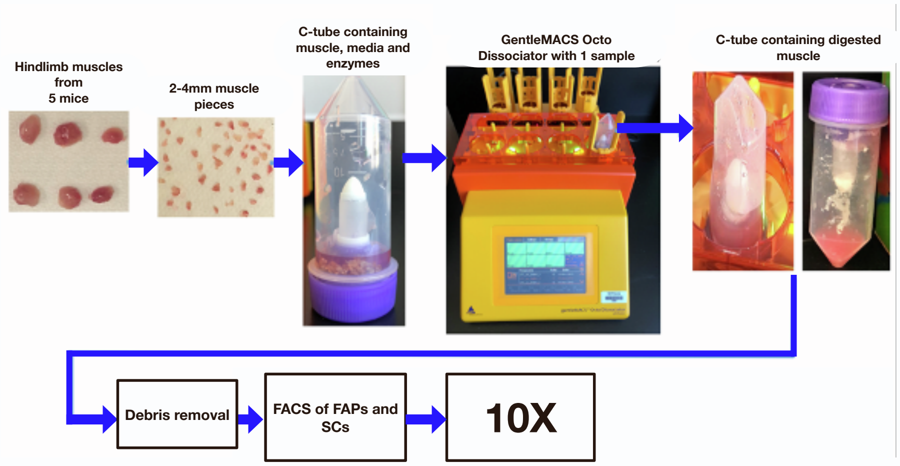

(b)

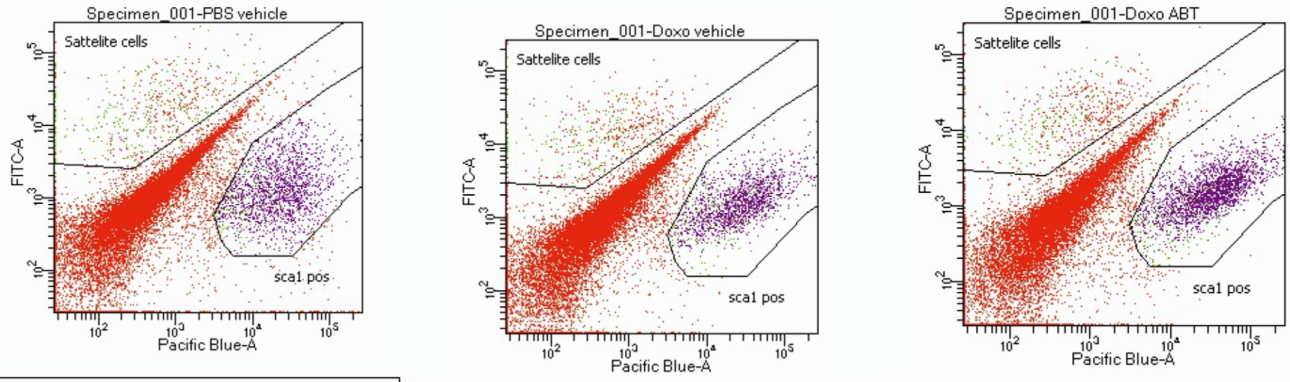

Tube: PBS vehicle

Population	#Events	%Parent	%Total
All Events	50,000	####	100.0
cells	33,147	66.3	66.3
fsc singlets	31,404	94.7	62.8
live nonhematopo	23,714	75.5	47.4
sca1 pos	1,825	7.7	3.6
FAP	1,635	89.6	3.3
Sattelite cells	587	2.5	1.2
P1	131	22.3	0.3
P2	242	41.2	0.5

Tube: Doxo vehicle

Population	#Events	%Parent	%Total
All Events	50,000	####	100.0
cells	36,121	72.2	72.2
fsc singlets	34,835	96.4	69.7
live nonhematopo	24,342	69.9	48.7
sca1 pos	1,646	6.8	3.3
FAP	1,427	86.7	2.9
Sattelite cells	546	2.2	1.1
P1	147	26.9	0.3
P2	195	35.7	0.4

Tube: Doxo ABT

Population	#Events	%Parent	%Total
All Events	50,000	####	100.0
cells	34,419	68.8	68.8
fsc singlets	33,278	96.7	66.6
live nonhematopo	20,618	62.0	41.2
sca1 pos	2,334	11.3	4.7
FAP	2,091	89.6	4.2
Sattelite cells	557	2.7	1.1
P1	175	31.4	0.4
P2	169	30.3	0.3

(c)

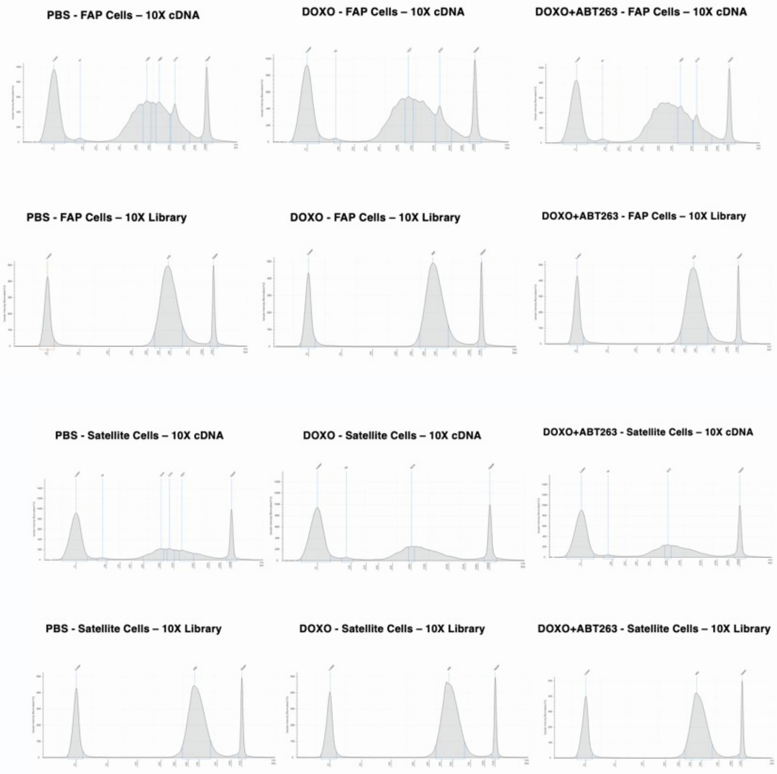

Supplementary Fig. 1 (related to Fig. 1). Workflow for single cell preparation (**a**) Workflow for FAPs and SCs sample preparation for single-cell RNA Seq on the 10X platform. (**b**) FACS gating strategy for isolating FAPs and SCs from mouse skeletal muscle. (**c**) 10X single-cell RNA seq-cDNA and library graphs for FAPs and SCs from each experimental group.

FAPs

DOXO vs PBS

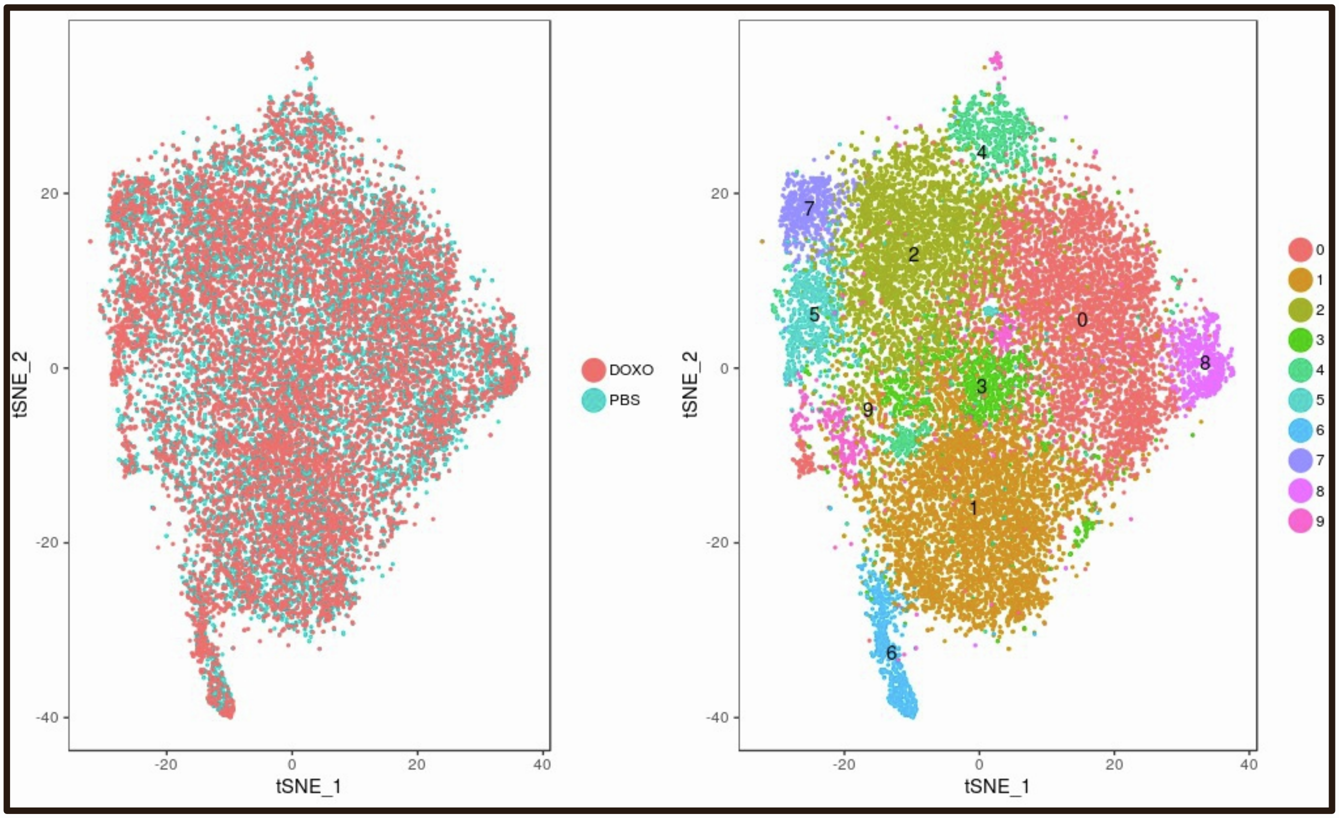

Supplementary Fig. 2 (related to Fig. 1).
t-SNE plots showing the embeddings (left) and clustering (right) of cells analyzed in FAPs DOXO vs PBS comparisons.

Supplementary Table 1 (related to Fig 1). Pairwise comparison of treated cells. Total number cells analyzed in each pairwise comparison of libraries, the clusters in which we observed significant expression changes in p16 and p21 between treatment groups, and corresponding figures for reference.

Cell Type	Comparison	Number of Cells	p16 regulation cluster	p21 regulation cluster	Corresponding figure panel
**FAPs**	DOXO vs. PBS	20,174	↑5	↑8	Supplementary Figure 2
	DOXO+ABT263 vs. DOXO	19,194	↓4	-	Figure 1b
**SCs**	DOXO vs. PBS	17,164	-	↑0, 5, 9; ↓4	Supplementary Figure 3a
	DOXO+ABT263 vs. DOXO	16,600	-	↑8; ↓3, 4, 5, 9	Supplementary Figure 3b

Supplementary Table 2 (related to Fig 1). Cell counts, median, and mean reads per cell for each library and cell type

Cell Type	Library	Cell Count	Mean Reads per Cell	Median Genes per Cell
**FAPs**	PBS	10,032	12,237	930
	Doxo	10,142	12,318	870
	Doxo+ABT	9,052	14,033	1,062
**SCs**	PBS	7,372	17,151	503
	Doxo	9,792	12,714	438
	Doxo+ABT	6,808	17,870	489

SCs

DOXO vs PBS

(a)

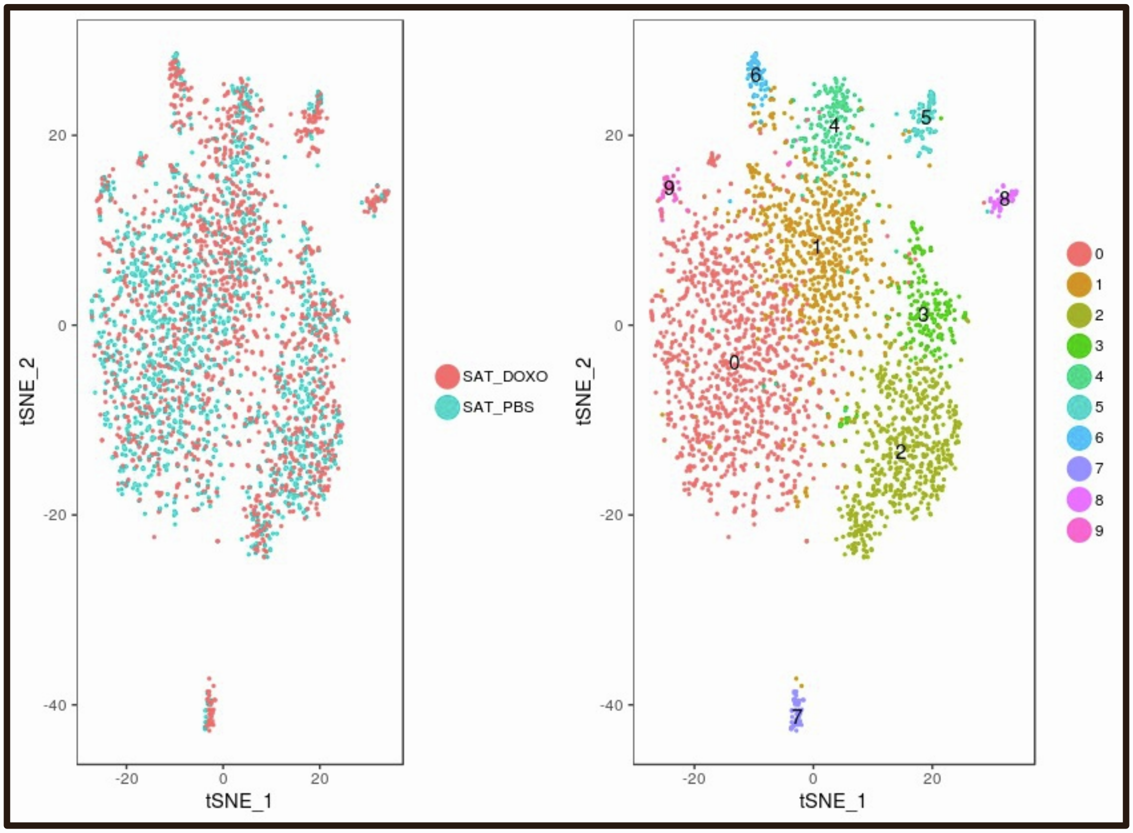

DOXO+ABT vs DOXO

(b)

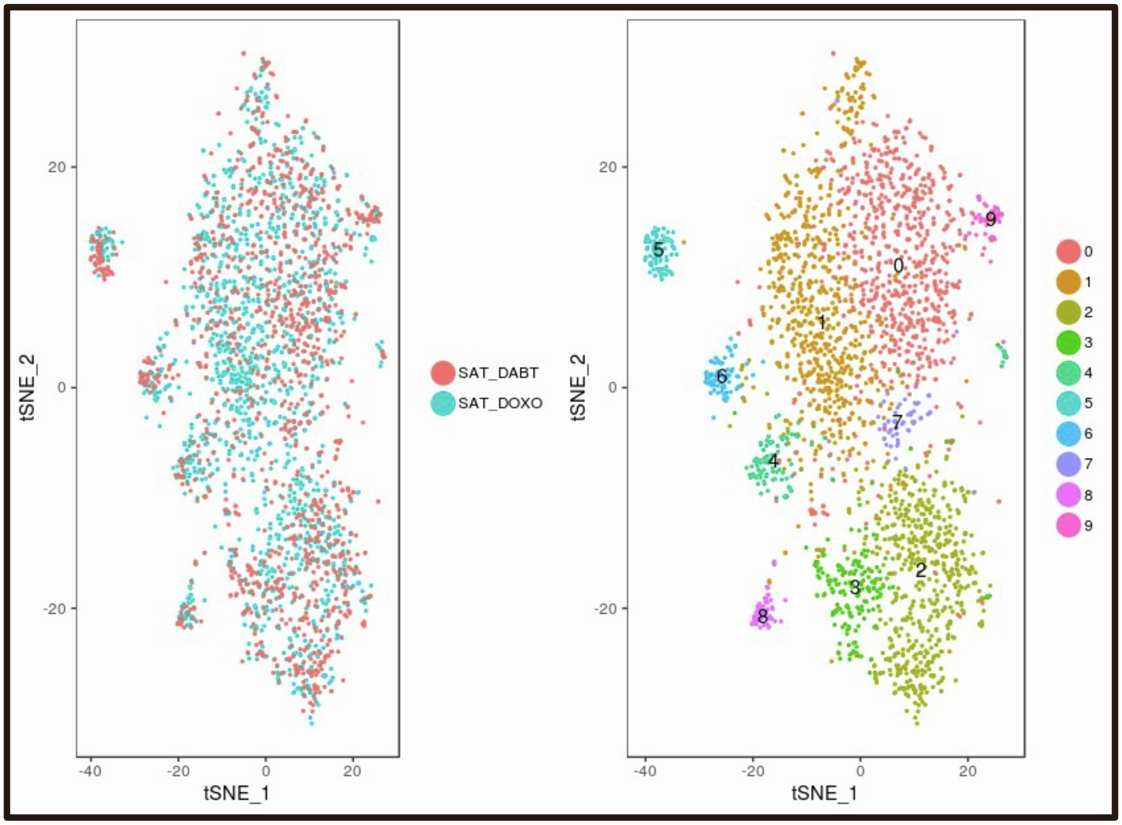

Supplementary Fig. 3 (related to Fig. 1). t-SNE plots showing the embeddings (left) and clustering (right) of cells analyzed in (**a**) SC DOXO vs PBS and (**b**) DOXO+ABT vs DOXO comparisons.

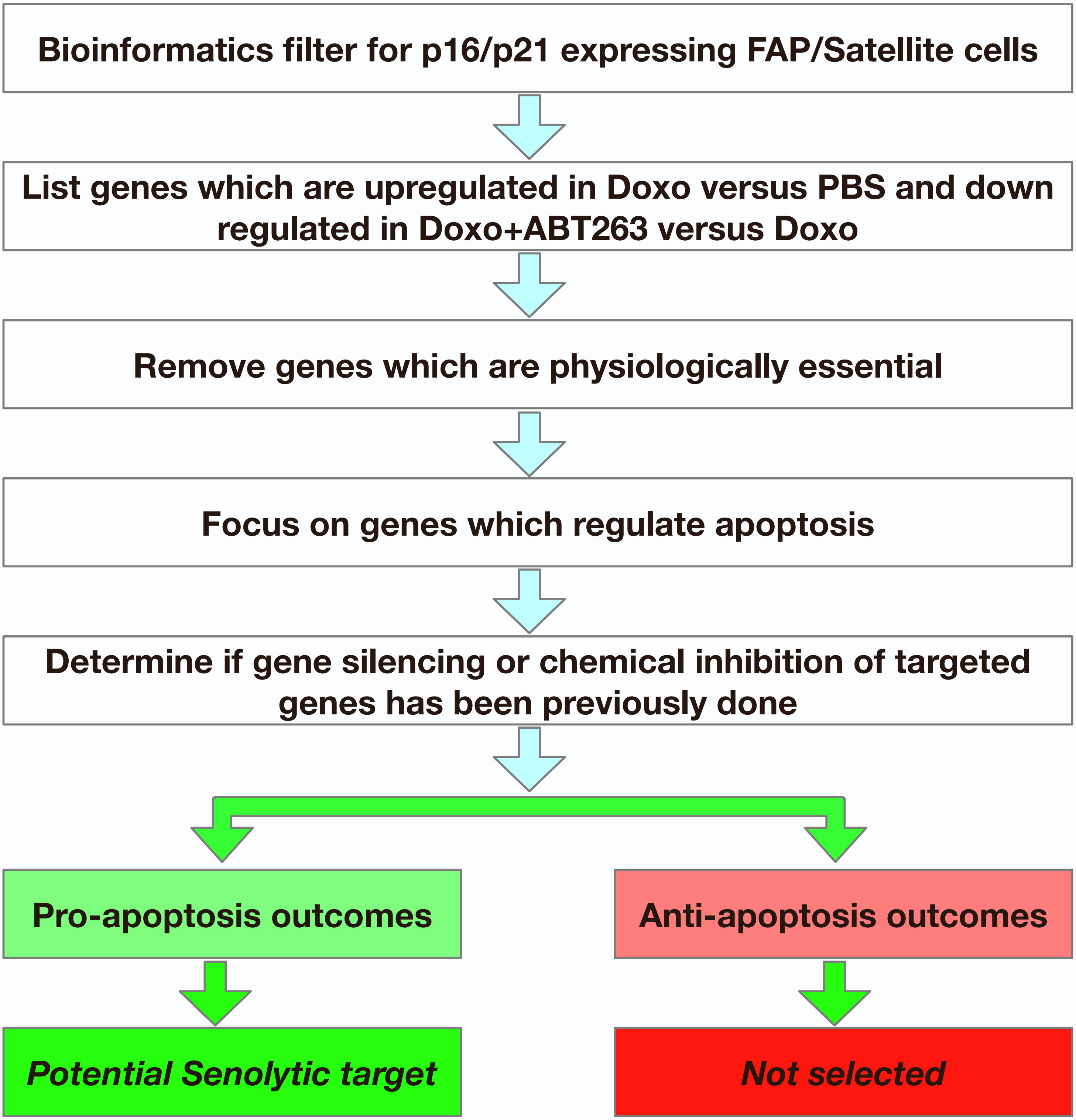

Supplementary Fig. 4 (related to Fig. 2). Strategy to identify potential senolytic target genes from FAP and SC single-cell RNA Seq data.

Supplementary Table 5 (related to Fig 2). Potential senolytic target genes.
Identified by single-cell RNA-seq analysis of senescent and non-senescent FAPs and Satellite Cells (SC) from mouse skeletal muscles

Cell Type	#	Gene Name	Full name
**FAPs**	1	Hmox1	Heme oxygenase 1
	2	Col1a1	Collagen type I alpha 1 chain
	3	Pltp	Phospholipid transfer protein
	4	Srxn1	Sulfiredoxin 1
**SCs**	5	Cryab	Crystallin alpha B
	6	Rgcc	Regulator of cell cycle
	7	Rnf7	Ring finger protein 7
	8	Fxyd3	FXYD domain containing ion transport regulator 3
	9	Itgb8	Integrin subunit beta 8
	10	Cav1	Caveolin 1

Supplementary Table 6 (related to Fig 3). Antibodies for FAP and SC isolation by FACS

Antibody	Clone	Conjugate	Dilution	Source	Cat.#
anti-CD31	MEC13.3	Alexa Fluor® 488	1:200	BioLegend	102514
anti-CD45	30-F11	Alexa Fluor® 488	1:200	BioLegend	103122
anti-Sca-1	D7	PE	1:80	Invitrogen	12-5981-82
anti-PDGFRa	Polyclonal	PE	1:80	R&D	FAB1062P
anti-VCAM1	429 (MVCAM.A)	PE/Cy7	1:40	BioLegend	105720

Supplementary Table 7 (related to Fig 3): IC50 of senolytics on mouse and human cell types

Cell type	days in culture	Inhibitor	IC50 (µM)
**FAPs**	3	ABT263	14.7
**FAPs**	7	ABT263	7.5
**FAPs**	3	25HC	1.7
**FAPs**	7	25HC	1.2
**FAPs**	7	OB24	261.4
**SCs**	7	ABT263	14.1
**SCs**	7	25HC	3.7
**HSMM**	3	ABT263	15.2
**HSMM**	3	25HC	7.2
**HSMM**	7	ABT263	9
**HSMM**	7	25HC	3.7

Supplementary Table 8 (related to Figs 5-9). Specific primer sequences for quantitative RT-PCR

Species	Genes	Forward (5′-3′)	Reverse (5′-3′)
Mouse	ß-Actin	CTAAGGCCAACCGTGAAAAG	ACCAGAGGCATACAGGGACA
	p16	GAACTCTTTCGGTCGTACCC	GTTCGAATCTGCACCGTAGT
	p21	ACGGGACCGAAGAGACAAC	CAGATCCACAGCGATATCCA
	Cryab	GTTCTTCGGAGAGCACCTGTT	GAGAGTCCGGTGTCAATCCAG
	Hmox1	GCCGAGAATGCTGAGTTCATG	TGGTACAAGGAAGCCATCACC
	Pltp	CTGACGCATGGCCAAGAAGA	CATGGCAGAGTCAAAGAAGA
	Rnf7	ACCCTGCGTCCTTTCTTCG	GGCACAGGTATCGCACTCAA
	Srxn1	GACGTCCTCTGGATCAAAG	GCAGGAATGGTCTCTCTCTG
	Rgcc	CGCCACTTCCACTACGAGG	CAGCAATGAAGGCTTCTAGCTC
	Itgb8	TGGCCCTTTATTCCCGTGAC	GGGTGGATACTAATGTATGGCGA
	Col1a1	CTGACGCATGGCCAAGAAGA	ATACCTCGGGTTTCCACGTC
	Cav1	GCGACCCCAAGCATCTCAA	ATGCCGTCGAAACTGTGTGT
	Il6	CTCTGGGAAATCGTGGAAAT	CCAGTTTGGTAGCATCCATC
	Mmp2	TCTGCGATGAGCTTAGGGAAAC	GACATACATCTTTGCAGGAGACAAG
	Timp2	GCATCACCCAGAAGAAGAGC	GTCCATCCAGAGGCACTCAT
	Tgfb3	TTGGGATATCGTTAGAGGCG	AAGAAGGAAGGCAGGAGGAG
Human	ß-ACTIN	CGTCATACTCCTGCTTGCTG	CGTCATACTCCTGCTTGCTG
	P16	CTACTGAGGAGCCAGCGTCT	CTGCCCATCATCATGACCT
	CRYAB	CTTTGACCAGTTCTTCGGAG	CCTCAATCACATCTCCCAAC
	HMOX1	ACTGCGTTCCTGCTCAACATC	GCTCTGGTCCTTGGTGTCATG

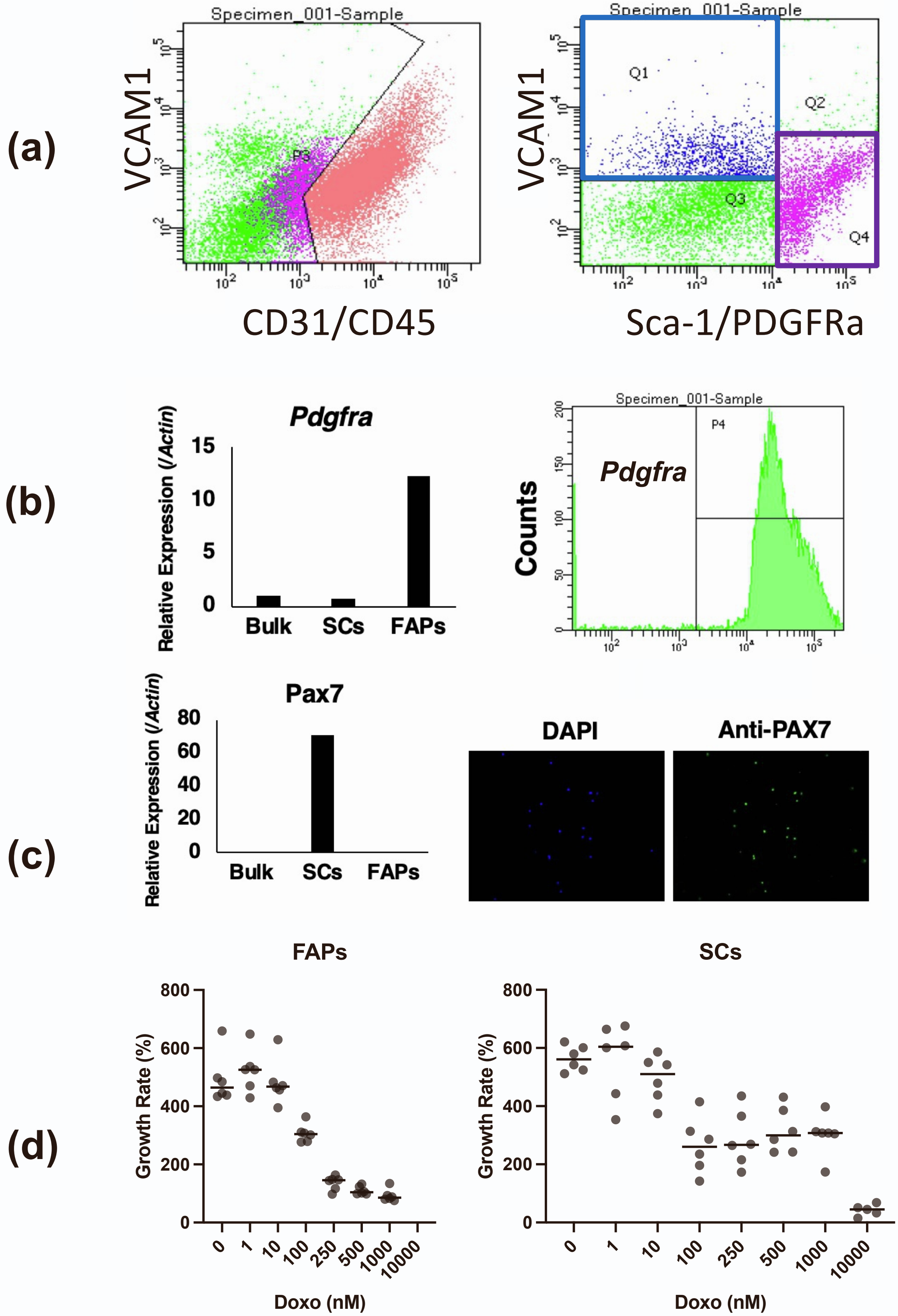

Supplementary Fig. 5 (related to Fig. 3). FAP and SC isolation for primary culture. (a) FACS gating strategy for isolating FAPs (purple: CD31-, CD45-, VCAM1- and Sca1+/PDGFR+) and SCs (blue: CD31-, CD45-, Sca1-, PDGFRa- and VCAM1+) from skeletal muscle. (**b**) (left) Expression analysis of *Pdgfra* in bulk populations, isolated SCs and FAPs by qRT-PCR. mRNA levels were normalized to *actin* mRNA. (Right) Reanalysis of FAP population by flow cytometry. (**c**) Expression analysis of Pax7 in bulk populations (Bulk), isolated SCs and FAPs. mRNA levels were normalized to *actin* mRNA. (right) PAX7 Immunostaining of SC population. Cells were fixed and stained with DAPI (blue) and anti-PAX7 (green). (**d**) Optimization of Doxo concentration for use in FAPs and SCs. The growth rate for 72 h was measured by cell counting.

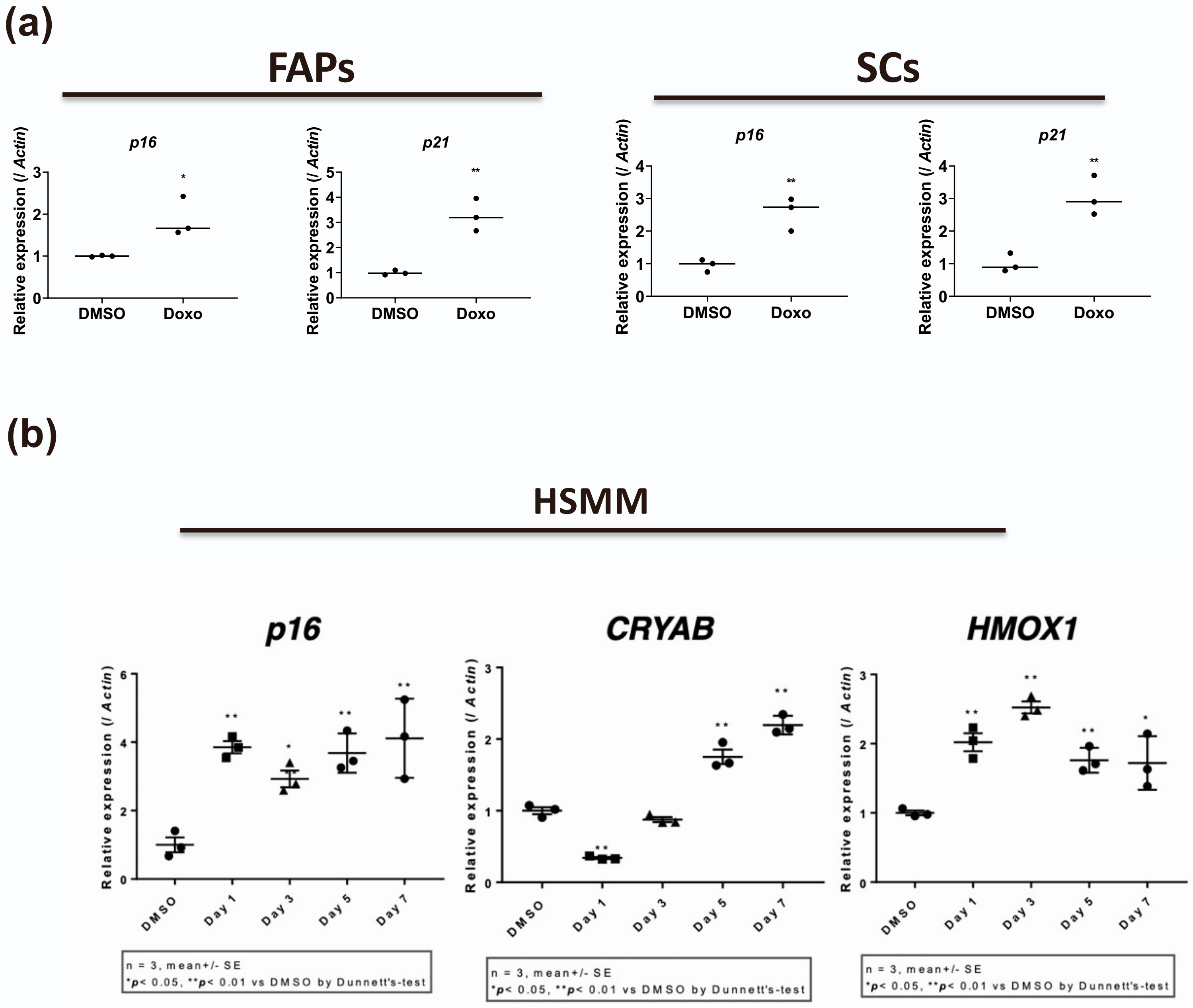

Supplementary Fig. 6 (related to Fig. 3.| Expression of senescence marker genes in FAPs, SCs and HSMMs after Doxo treatment. (a) Expression analysis of senescent marker genes in FAPs and SCs. (b) Expression analysis of senescent marker and target genes in HSMMs. RNA was collected at 7 d (**a**) or the indicated times, or **(b)** after replacing media containing Doxo. mRNA levels were quantified by qRT-PCR, and normalized *actin* mRNA. The average value of DMSO was set at 1. *p*-values versus DMSO were obtained using unpaired two-tailed Student's t-test (a) or Dunnett's multiple comparison test (b). *$p< 0.05$, **$p< 0.01$, ***$p< 0.001$ and ****$p< 0.0001$.

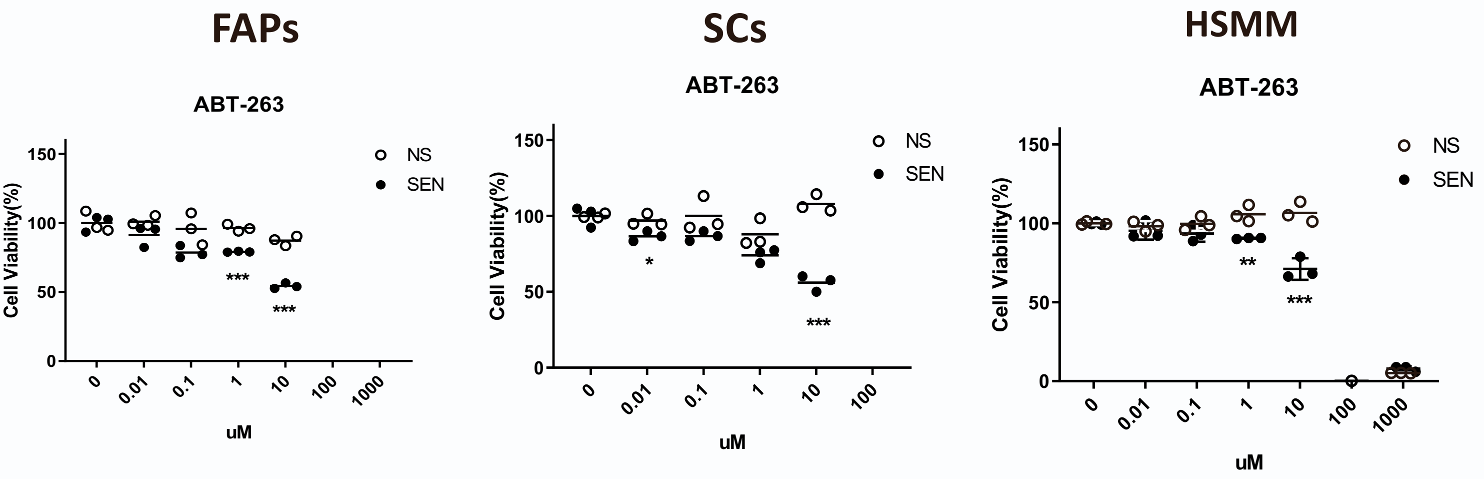

Supplementary Fig. 7 (related to Fig. 4). Effects of ABT-263 on viability of FAPs, SCs and HSMMs. Cells were cultured for 24 h, treated with Doxo for 24 h, then treated with ABT-263 at the indicated concentrations. Cell viability was analyzed at day 3 (HSMMs) or 5 (FAPs and SCs) for non-senescent (NS) and senescent cells (SEN). Black and white dots indicate NS and SEN, respectively. The average value of 0 M for each group was set at 100%. *p*-values versus NS are obtained using unpaired two-tailed Student's t-test. *$p < 0.05$, **$p < 0.01$, ***$p < 0.001$.

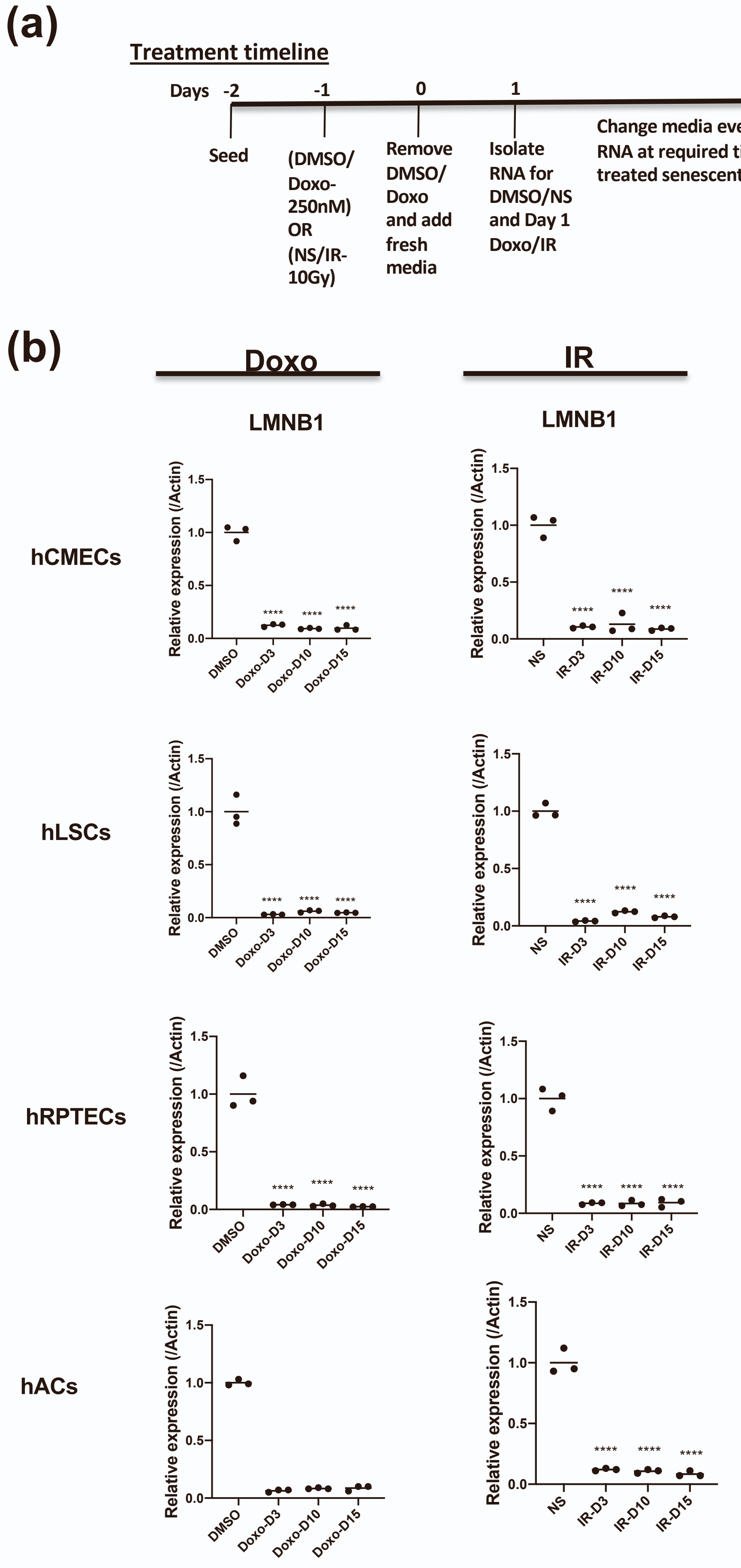

Supplementary Fig. 8 (related to Fig. 6). LMNB1 expression in response to Doxo or IR (**a**) Experimental timeline. (**b**) Doxo or IR reduced LMNB1 mRNA in hCMECs, hLSCs, hRPTECs, and hACs. Senescence was induced by either 250 nM Doxo for 24 h or 10 Gy X-rays. qRT-PCR was performed on RNA samples from DMSO/ Doxo treated or NS/IR-treated non-senescent or senescent (SEN) cells at indicated times. mRNA levels were normalized to *actin* mRNA. The average values of DMSO or NS was set at 1. For qRT-PCR: mean +/- SE, n =4, *$p < 0.05$, **$p < 0.01$, ***$p < 0.001$, ****$p < 0.0001$ by Dunnett's multiple comparisons test vs DMSO or NS.

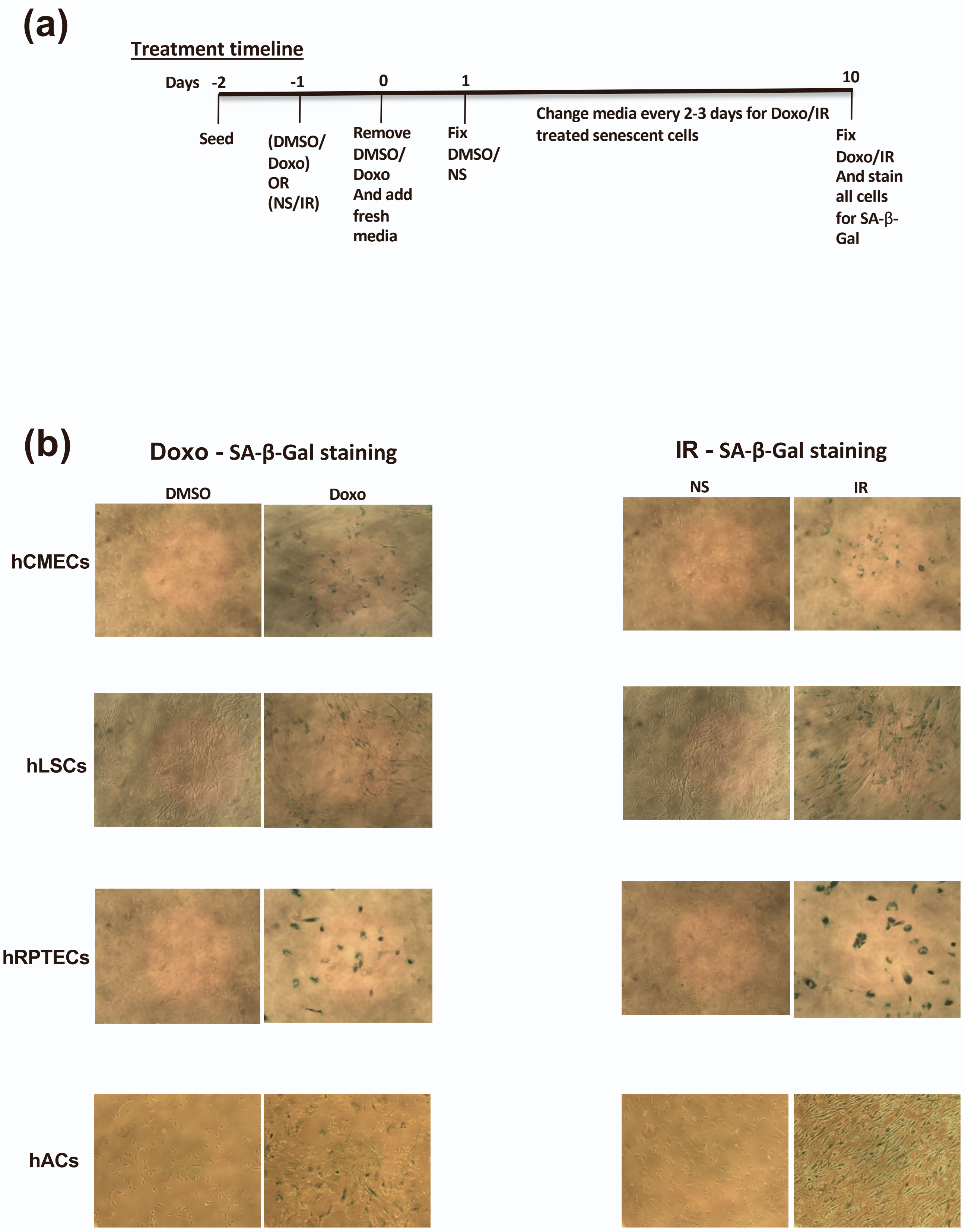

Supplementary Fig. 9 (related to Fig. 6) | SA-ß-Gal staining in diverse cell types (**a**) Experimental timeline. (**b**) Doxo or Ionizing Radiation(IR) induced SA-β-gal in hCMECs, hLSCs, hRPTECs and hACs. 10 d after DMSO/Doxo or Non-Senescent (NS)/IR treatment, SA-β-gal staining was performed. The majority of cells were SA-ß-Gal positive after Doxo or IR.

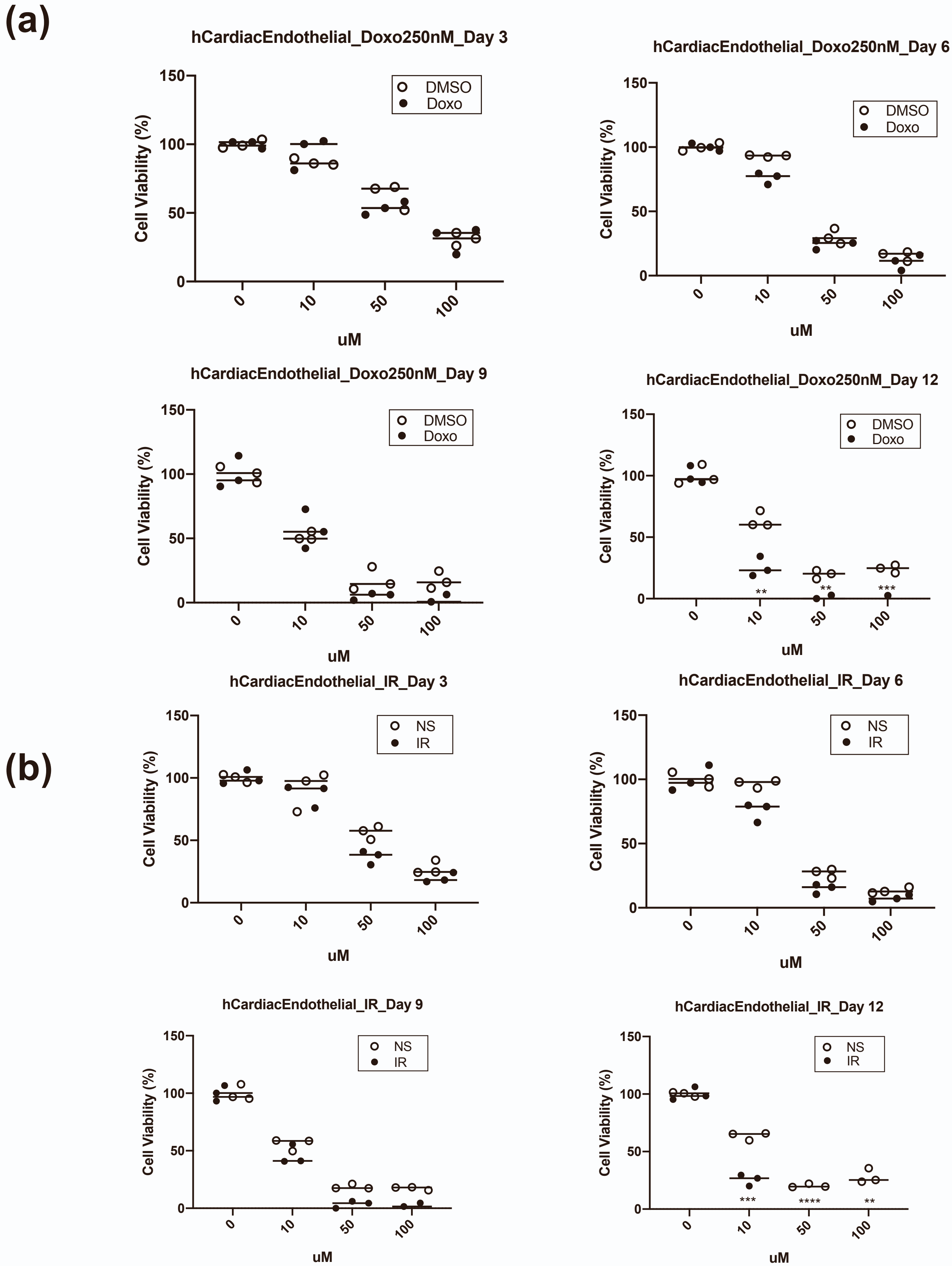

Supplementary Fig. 10 (related to Fig. 6-7). 25HC treatment of human cardiac endothelial cells (**a**) 25HC effects on DMSO- or Doxo-treated hCMECs at various times. Cells were treated with DMSO or 250 nM Doxo for 24 h. 25HC treatment was started 24 h after DMSO or Doxo treatment. Cell viability was measured every 3–5 days until 15 days. (**b**) 25HC effects on NS or IR-treated hCMECs at various times. Cells were treated with either NS or 10-Gy IR. 25HC treatment was started 24 h later. Cell viability was measured every 3–5 days until 15 days. For cell viability assays: mean +/- SE, n =3, ***p*< 0.01, ****p*<0.001, *****p*<0.0001 vs 0 mM by student's t-test.

(a)

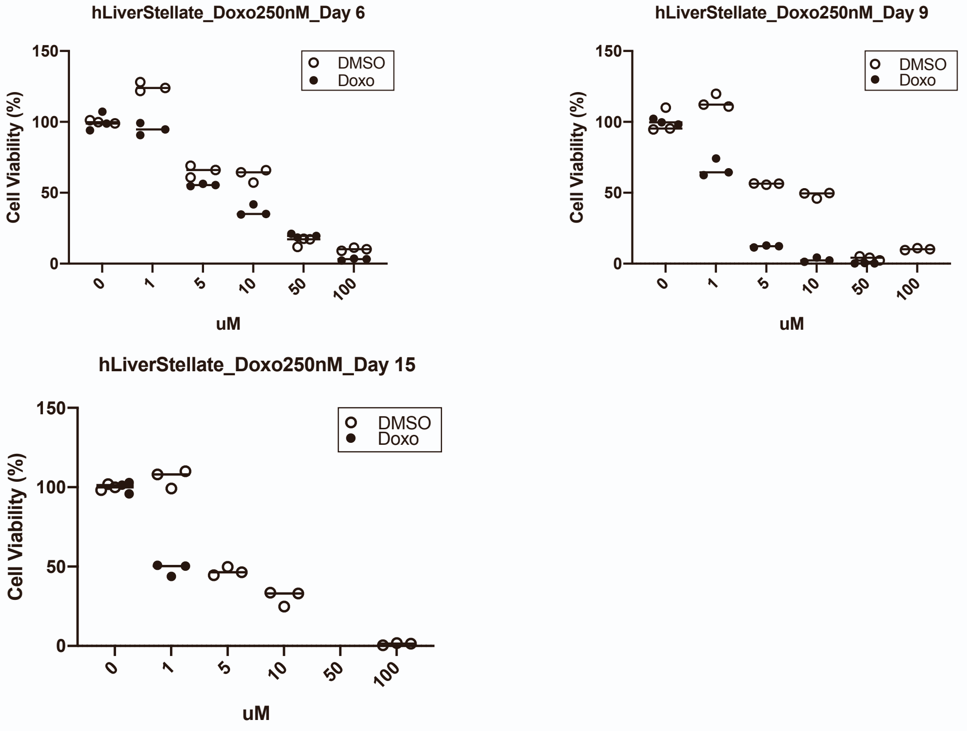

(b)

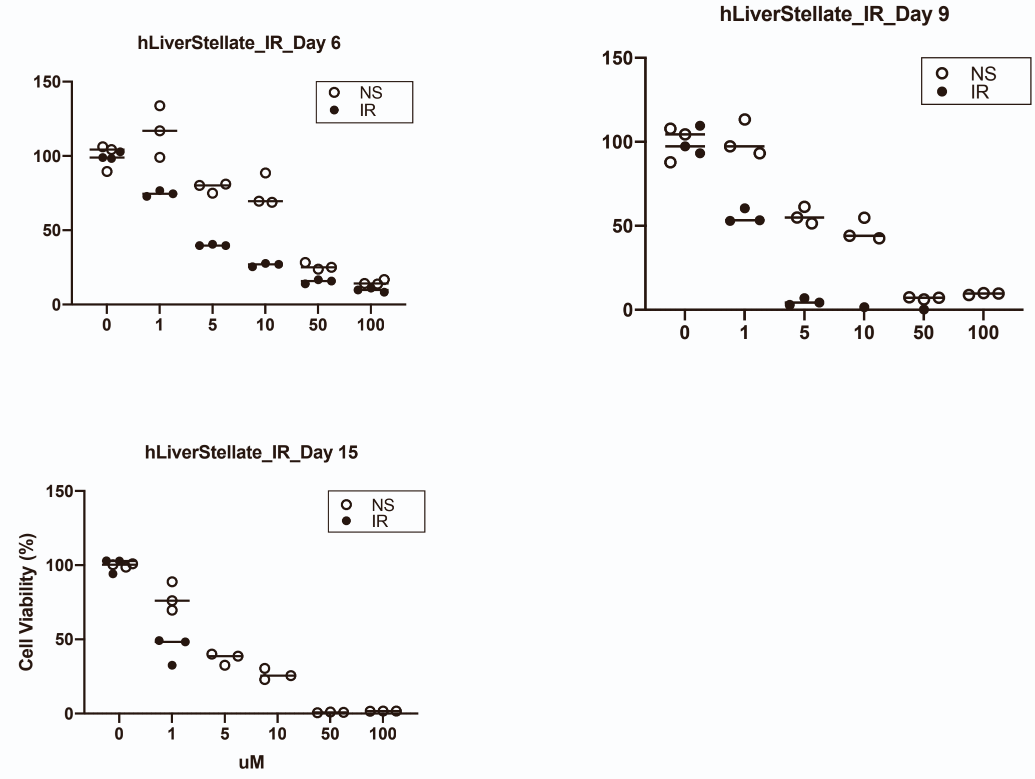

Supplementary Fig. 11 (related to Fig. 7). 25HC treatment of human liver stellate cells (**a**) 25HC effects on DMSO- or Doxo-treated hLSCs at various times. Cells were treated with either DMSO or 250 nM Doxo for 24 h. 25HC treatment was started 24 h later. Cell viability was measured every 3-–5 days until 15 days. (**b**) 25HC effects on NS or IR-treated hLSCs at various times. Cells were either NS or SEN. 25HC treatment was started 24 h later. Cell viability was measured every 3–5 days until 15 days. For cell viability assays: mean +/- SE, n =3, ***p*< 0.01, ****p*<0.001, *****p*<0.0001 vs 0 mM by student's t-test.

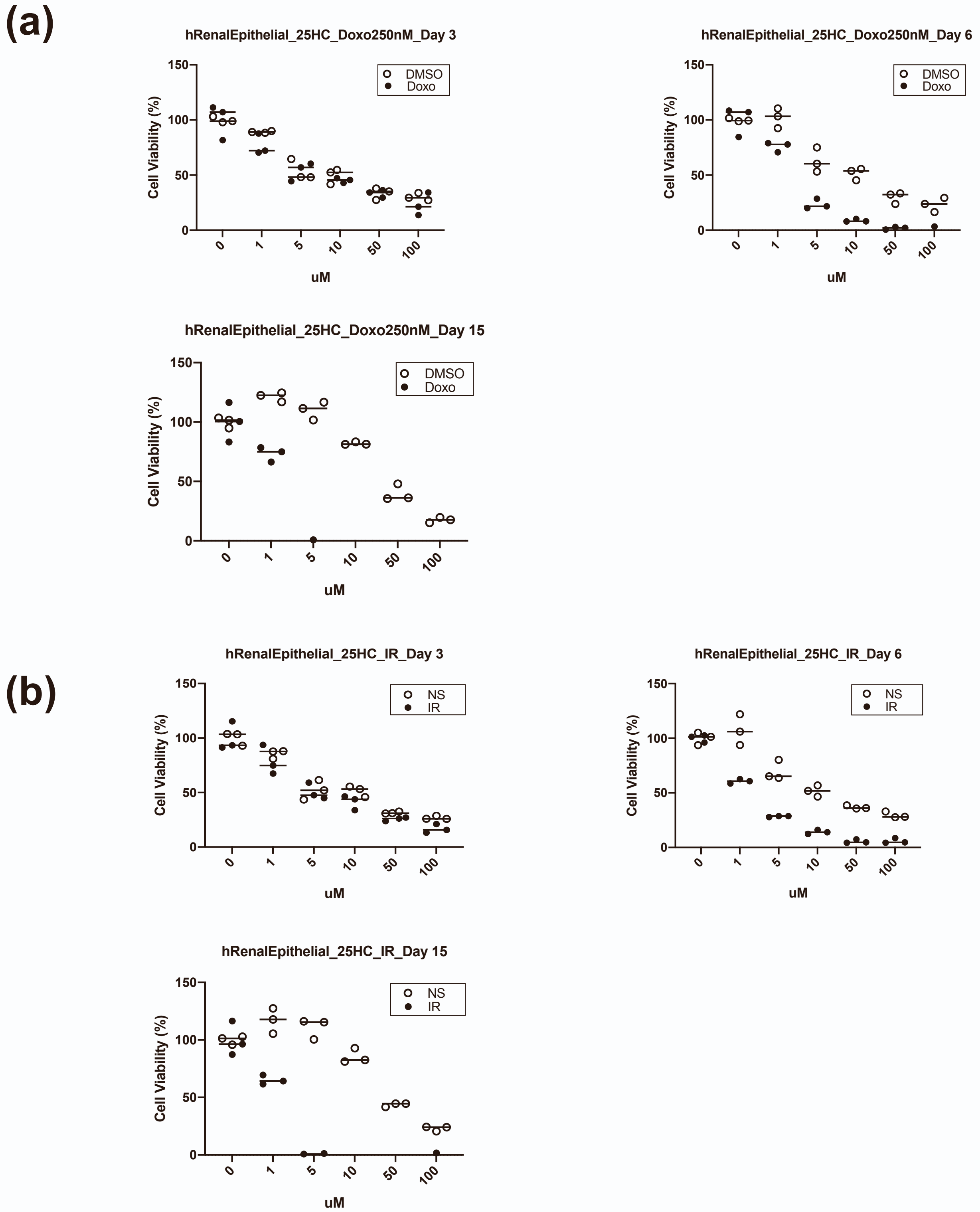

Supplementary Fig. 12 (related to Fig. 7) | 25HC treatment of human renal epithelial cells (**a**) 25HC effects on DMSO- or Doxo-treated hRPTECs at various times. Cells were treated with DMSO or 250 nM Doxo for 24 h. 25HC treatment was started 24 h later. Cell viability was measured every 3–5 days until 15 days. (**b**) 25HC effects on NS or IR-treated hRPTECs at various times. Cells were NS or treated with 10-Gy X-rays. 25HC treatment was started 24 h later. Cell viability was measured every 3–5 days until 15 days. For cell viability assays: mean +/- SE, n =3, **p< 0.01, ***p<0.001, ****p<0.0001 vs 0 mM by student's t-test.

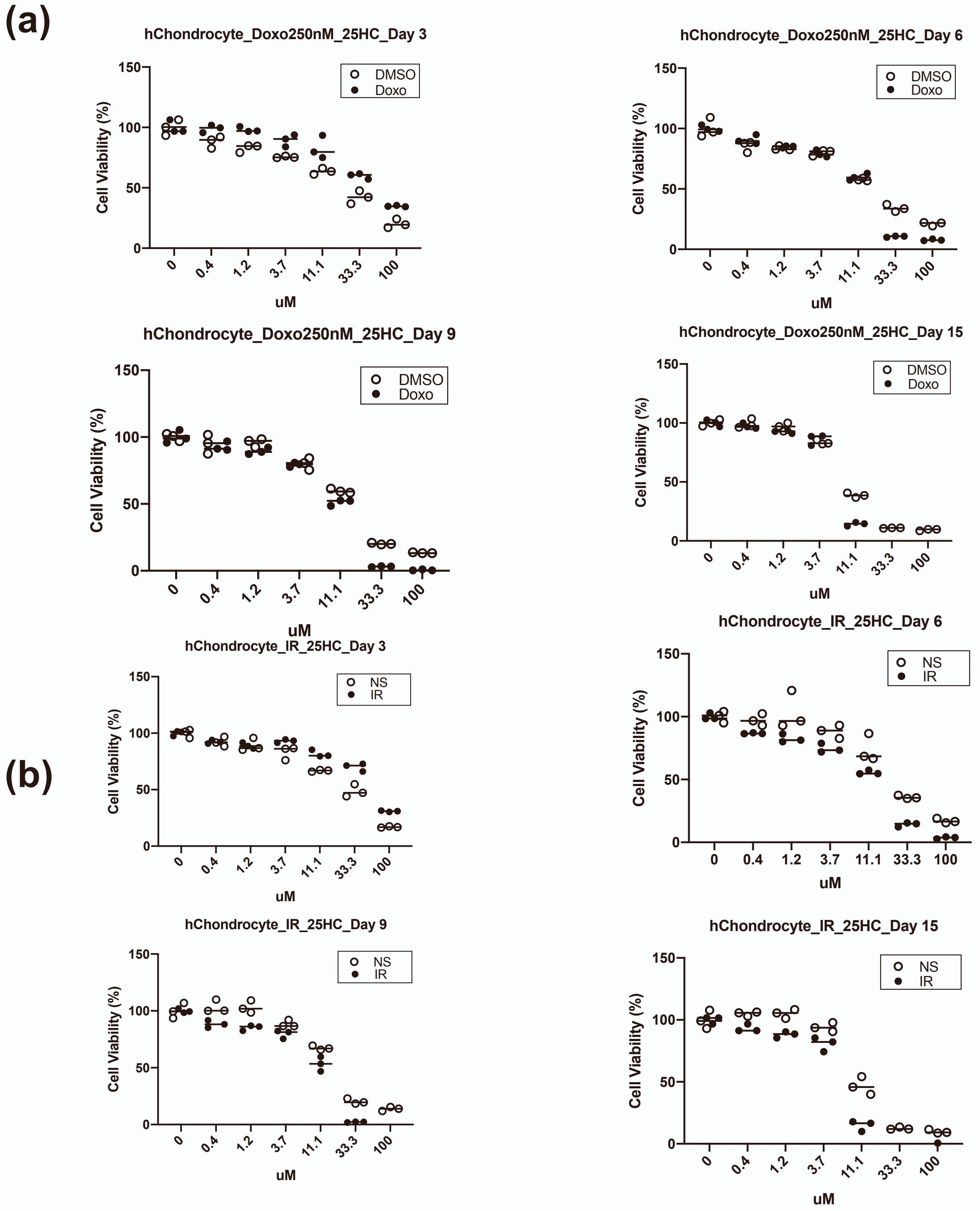

Supplementary Fig. 13 (related to Fig. 7). 25HC treatment of human articular chondrocytes (**a**) 25HC effects on DMSO- or Doxo-treated hACs at various times. Cells were treated with DMSO or 250 nM Doxo for 24 h. 25HC treatment was started 24 h later. Cell viability was measured every 3–5 days until 15 days. (**b**) 25HC effects on NS or IR-treated hACs at various times. Cells were NS or treated with 10-Gy X-rays. 25HC treatment was started 24 h later. Cell viability was measured every 3–5 days until 15 days. For cell viability assays: mean +/- SE, n =3, $^{**}p < 0.01$, $^{***}p<0.001$, $^{****}p<0.0001$ vs 0 mM by student's t-test.

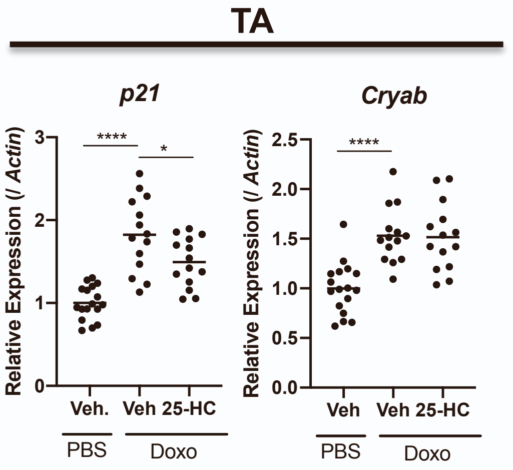

Supplementary Fig. 14 (related to Fig. 8). Expression of *Cdkn2a, Cdkn1a* and *CryAB* in visceral fat, tibialis anterior (TA) muscle, kidney, liver, lung, heart and skin of Doxo-treated mice. qRT-PCR was performed on RNA from the tissue above, and mRNA levels were normalized to *actin* mRNA. The average value of the vehicle group was set at 1. *p*-values versus PBS-Vehicle or Doxo-Vehicle are obtained using Tukey's multiple comparisons test. *$p < 0.05$, ***$p < 0.001$ and ****$p < 0.0001$.

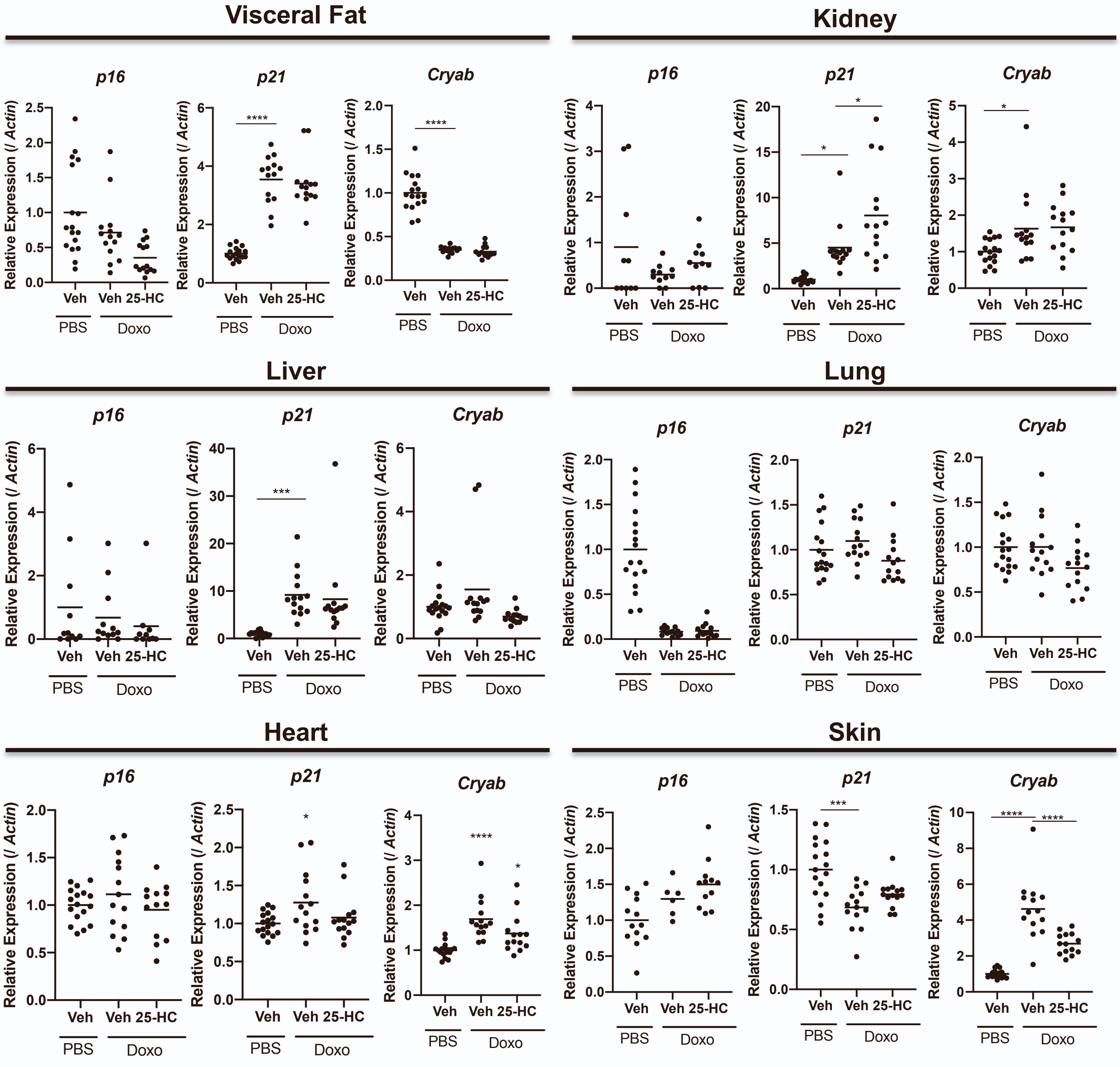

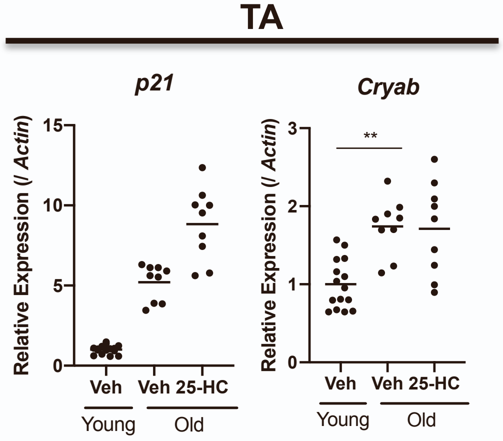

Supplementary Fig. 15 (related to Fig. 9). Expression of *Cdkn2a, Cdkn1a* and *CryAB* in visceral fat, tibialis anterior (TA) muscle, kidney, liver, lung, heart and skin of old male mice. qRT-PCR was performed on RNA from the tissues above, and mRNA levels were normalized to *actin* mRNA. The average value of the young vehicle group was set at 1. *p*-values versus Young-Vehicle or Old-Vehicle were obtained using Tukey's multiple comparisons test. *$p < 0.05$, **$p < 0.01$, ***$p < 0.001$****$p < 0.0001$.

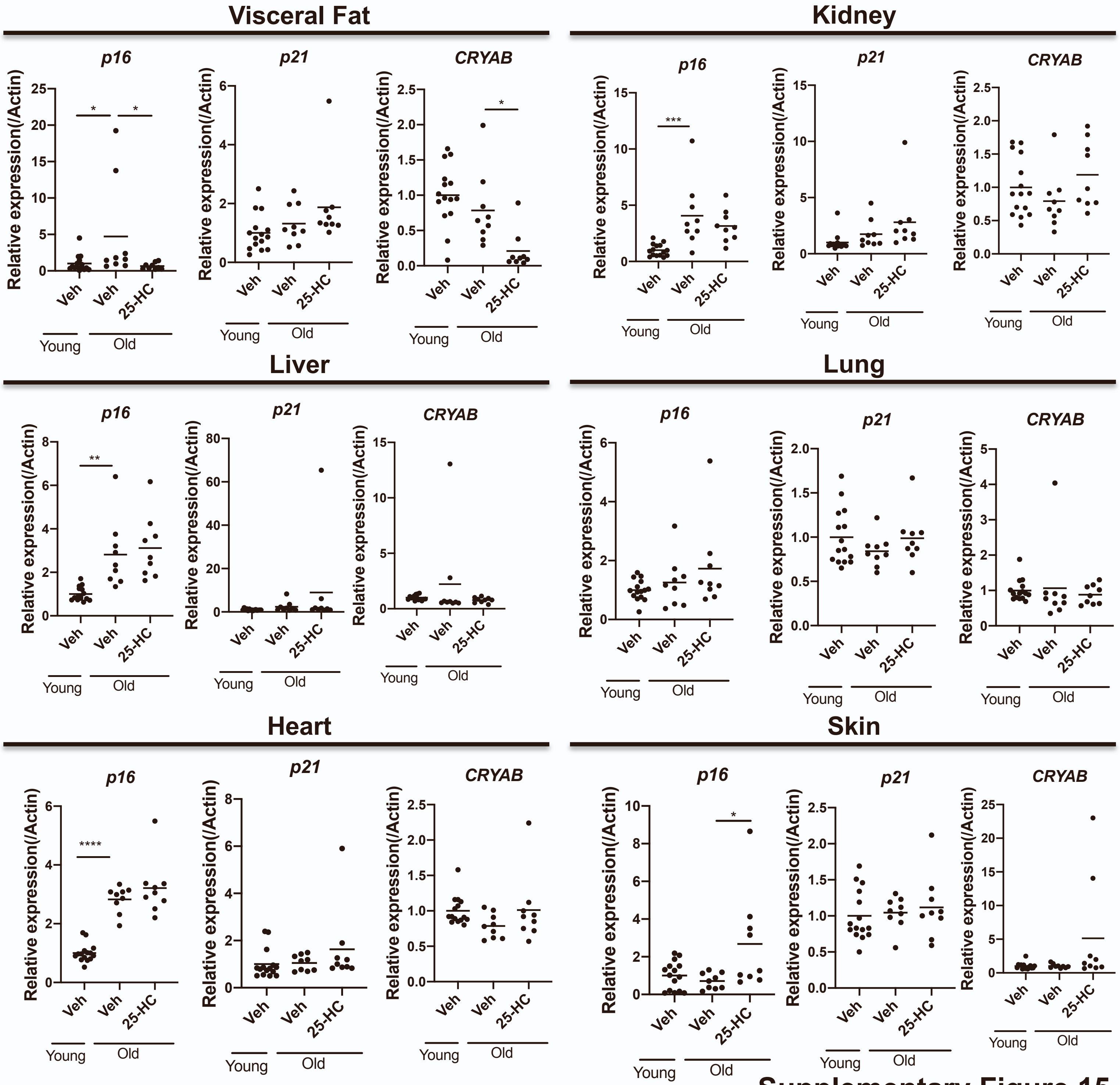

Supplementary Figure 15

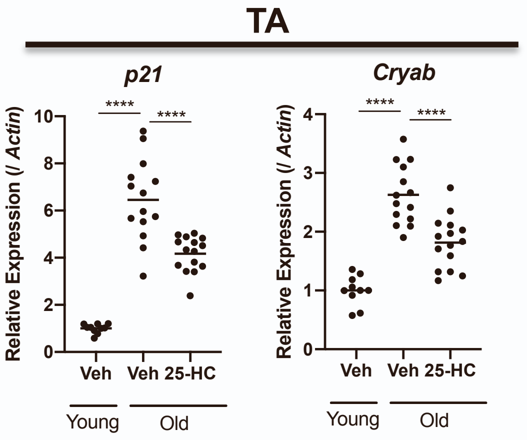

Supplementary Fig. 16 (related to Fig. 9). Expression of *Cdkn2a, Cdkn1a* and *CryAB* in visceral fat, tibialis anterior (TA) muscle, kidney, liver, lung, heart and skin of old female mice. qRT-PCR was performed on RNA from the tissues above, and mRNA levels were normalized to *actin* mRNA. The average value of the young vehicle group was set at 1. *p*-values versus Young-Vehicle or Old-Vehicle are obtained using Tukey's multiple comparisons test. *$p<$ 0.05, **$p<$ 0.01, ***$p<$ 0.001****$p<$ 0.0001.

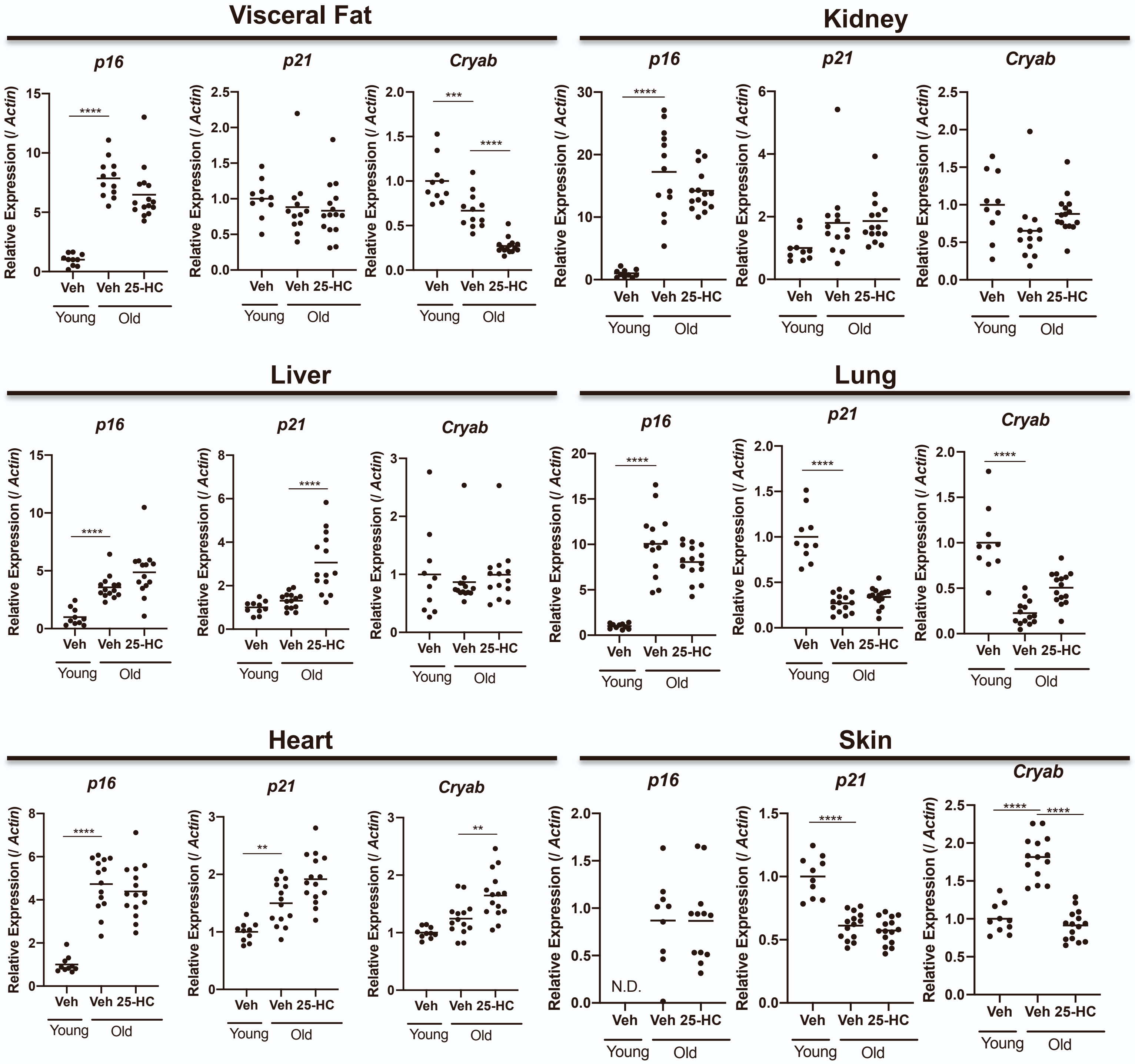

Supplementary Figure 16

Supplementary Fig. 17 (related to Fig. 8). Expression of SASP factors *Il6*, *Mmp2*, *Timp2* and *Tgfb3* in Gastroc and soleus muscles of Doxo-treated mice. qRT-PCR was performed on RNA from the muscles. mRNA levels were normalized to actin mRNA. The average value of the vehicle group was set at 1. *p*-values versus PBS-Veh or Doxo-Veh are obtained using Tukey's multiple comparisons test. ****$p < 0.0001$.

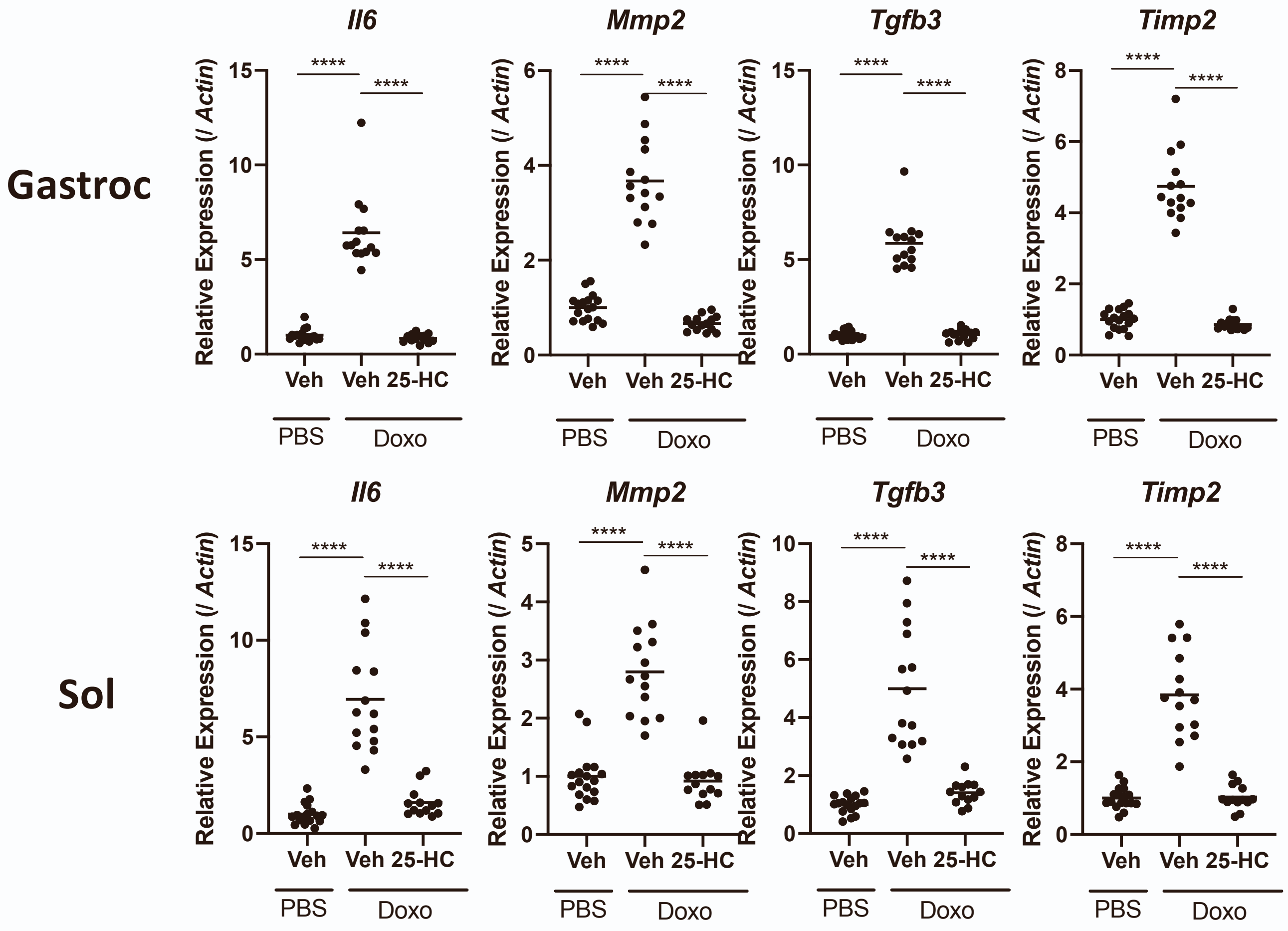

Supplementary Figure 17

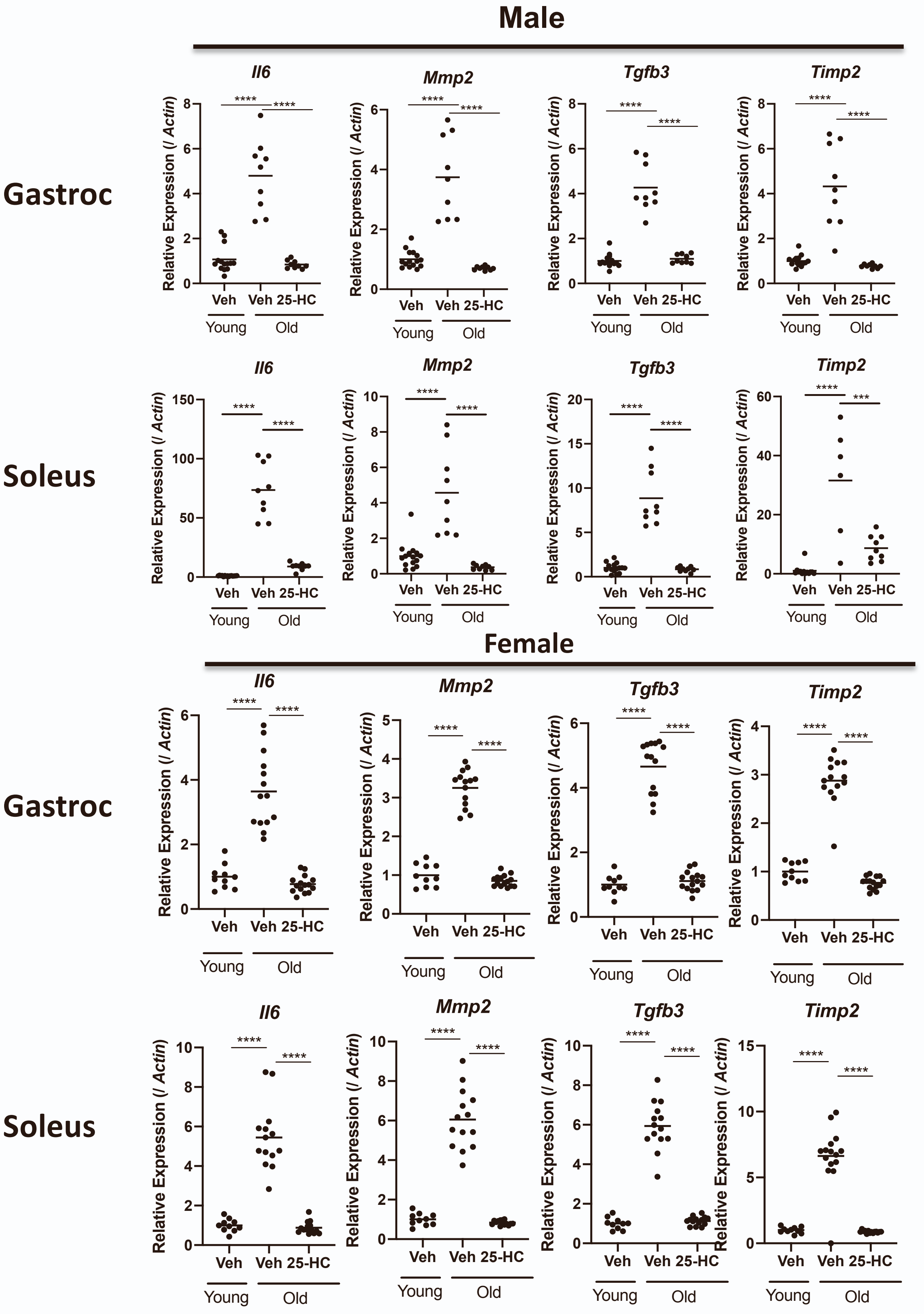

Supplementary Fig. 18 (related to Fig. 9). Expression of SASP factors *Il6*, *Mmp2*, *Timp2* and *Tgfb3* in Gastroc and soleus muscles of 25HC treated aged mice. qRT-PCR was performed on RNA from the muscles. mRNA levels were normalized to *actin* mRNA. The average value of the vehicle group was set at 1. *p*-values versus Young-Vehicle or Old-Vehicle is obtained using Tukey's multiple comparisons test. ****p*< 0.001 and *****p*< 0.0001.
